# Inequalities in developing multimorbidity over time: A population-based cohort study from an urban, multi-ethnic borough in the United Kingdom

**DOI:** 10.1016/j.lanepe.2021.100247

**Published:** 2021-11-04

**Authors:** Alessandra Bisquera, Ellie Bragan Turner, Lesedi Ledwaba-Chapman, Rupert Dunbar-Rees, Nasrin Hafezparast, Martin Gulliford, Stevo Durbaba, Marina Soley-Bori, Julia Fox-Rushby, Hiten Dodhia, Mark Ashworth, Yanzhong Wang

**Affiliations:** a King's College London, School of Population Health & Environmental Sciences, London, UK; b NIHR Biomedical Research Centre, Guy's and St Thomas’ NHS Foundation Trust and King's College London, London, UK; c Outcomes Based Healthcare, Cavendish Square, London, UK

**Keywords:** Multimorbidity, Multi state markov chain, Probabilities, Long term conditions, LTC, Long term conditions, UK, United Kingdom, QOF, Quality Outcomes Framework, MCF, Mean cumulative function, IMD, Indices of Multiple Deprivation

## Abstract

**Background:**

Social and material deprivation accelerate the development of multimorbidity, yet the mechanisms which drive multimorbidity pathways and trajectories remain unclear. We aimed to examine the association between health inequality, risk factors and accumulation or resolution of LTCs, taking disease sequences into consideration.

**Methods:**

We conducted a retrospective cohort of adults aged 18 years and over, registered between April 2005 and May 2020 in general practices in one inner London borough (*n* = 826,936). Thirty-two long term conditions (LTCs) were selected using a consensus process, based on a definition adapted to the demographic characteristics of the local population. sThe development and resolution of these LTCs were examined according to sociodemographic and clinical risk factors (hypertension; moderate obesity (BMI 30·0–39·9 kg/m2), high cholesterol (total cholesterol > 5 mmol/L), smoking, high alcohol consumption (>14 units per week), and psychoactive substance use), through the application of multistate Markov chain models.

**Findings:**

Participants were followed up for a median of 4.2 years (IQR = 1·8 - 8·4); 631,760 (76%) entered the study with no LTCs, 121,424 (15%) with 1 LTC, 41,720 (5%) with 2 LTCs, and 31,966 (4%) with three or more LTCs. At the end of follow-up, 194,777 (24%) gained one or more LTCs, while 45,017 (5%) had resolved LTCs and 27,021 (3%) died. In multistate models, deprivation (hazard ratio [HR] between 1·30 to 1·64), female sex (HR 1·13 to 1·20), and Black ethnicity (HR 1·20 to 1·30; vs White) were independently associated with increased risk of transition from one to two LTCs, and shorter time spent in a healthy state. Substance use was the strongest risk factor for multimorbidity with an 85% probability of gaining LTCs over the next year. First order Markov chains identified consistent disease sequences including: chronic pain or osteoarthritis followed by anxiety and depression; alcohol and substance dependency followed by HIV, viral hepatitis, and liver disease; and morbid obesity followed by diabetes, hypertension, and chronic pain.

**Interpretation:**

We examined the relations among 32 LTCs, taking the order of disease occurrence into consideration. Distinctive patterns for the development and accumulation of multimorbidity have emerged, with increased risk of transitioning from no conditions to multimorbidity and mortality related to ethnicity, deprivation and gender. Musculoskeletal disorders, morbid obesity and substance abuse represent common entry points to multimorbidity trajectories.

**Research in context**

**Evidence before this study**

We searched PubMed, up to April 30, 2021, for published population-based cohort studies examining multimorbidity trajectories over time in adults 18 years and over. The search terms were “multimorbidity” and “longitudinal” or “trajectories”. The studies must include a follow-up time component, and consider the impact of age, socioeconomic or clinical risk factors to changes in multimorbidity progression. Existing cohort studies focus on the accumulation of diseases over time and assume unidirectional worsening disease trajectories.

**Added value of this study**

With the availability of resolved codes and dates of death, we were not only able to determine which subgroups of patients progressed (accumulates conditions, or dies) over the time, but also which ones were stable or remitted. We have quantified the likelihood of different multimorbidity trajectories in terms of both accumulation of diseases as well as disease type.

**Implications of all the available evidence**

Distinctive patterns for the development and accumulation of multimorbidity have emerged, with increased risk of transitioning from a healthy state to multimorbidity and mortality related to ethnicity, deprivation, and gender. In a young, multi-ethnic population, the common entry points to multimorbidity include chronic pain, hypertension, depression, and substance use.

Alt-text: Unlabelled box

## Introduction

Multimorbidity— often defined as the co-occurrence of 2 or more long term conditions (LTCs)— is highly prevalent among older people. In England, 54% of people aged 65 and over have multimorbidity and this is projected to increase to 68% by 2035.^1^ Multimorbidity is associated with lower quality of life and functional status and increased health care utilization and mortality.^2^, ^3^, ^4^, ^5^ Previous studies of multimorbidity progression were often cross-sectional, assessing dyads and triads of diseases or comparing the characteristics patients with and without multimorbidity using descriptive statistics.^1^^,^^6^, ^7^, ^8^ These analyses have increased understanding of the prevalence and clustering of diseases but provided less information about how multimorbidity develops over time between individuals and groups.

There has been increasing interest in longitudinal multimorbidity research to allow for a better understanding of disease sequences and trajectories, which may have important implications for clinical and population health intervention.^9^, ^10^, ^11^, ^12^ The Academy of Medical Sciences considers this area of work to be a research priority, especially whether trends and patterns differ between populations and subsets of the population.^13^ Modifiable socioeconomic factors and behaviours are key areas to target for short and long-term interventions, particularly in a young, deprived population. The rate of concurrent physical and mental health conditions, and the relationship between multimorbidity and low quality of life, higher health care costs, and mortality, affects this group more severely.^14^, ^15^, ^16^ For example, among incidences of single conditions, 22% were in the most deprived quintile and 19% in the least deprived and for dual conditions, 26% were in the most and 16% int the least deprived. Deaths in participants were higher in the most deprived group at 22% (with no conditions) to 33% (with triple conditions) versus 19% (no conditions) to 17% (with triple conditions in the least deprived.^14^ ther risk factors for a faster rate of disease accumulation, such as obesity, are attenuated in people living in deprived areas.^17^

Existing cohort studies focus on the accumulation of diseases over time, without specifying the type of disease,^11^^,^^18^^,^^19^ or they may account for disease type but assume that the effect of time is constant on the movement between states.^20^ Some studies attempt to examine both accumulation and disease type, however they focus on only a small number of diseases, to keep the number of possible states of progression at a manageable level.^21^, ^22^, ^23^, ^24^ Newer analytical methods, including multistate Markov models, make it possible to incorporate multiple disease states in a probabilistic framework, but these have not been widely used in multimorbidity research.

Previous multimorbidity studies generally assume unidirectional worsening disease trajectories and disregard disease resolution. The aims of this study were 1) to investigate the association between health inequality risk factors and accumulation or resolution of LTCs; 2) to identify groups with rapid and slow progress of LTCs/diseases, and 3) to estimate probabilities of acquiring different LTCs.

## Methods

### Study design, setting and participants

This study is a retrospective cohort with anonymised coded data on all eligible patients aged 18 years and over between 1/4/2005 to 1/5/2020 extracted from electronic health records (EHRs) held in primary care. The EHR is the longitudinal clinical record of healthcare for all patients registered with a general practice in the UK. The data is entered by health care professionals at the practice where the patient is registered. Data is extracted quarterly in arrears, based on coded data such as Read, SNOMED-CT and Medication codes. The population sample consisted of patients registered at general practices (*n* = 41) in an inner-city borough in south London, excluding (3·2%) who opted out of anonymised data sharing for research. The data reflects a deprived, multi-ethnic, youthful population. The prevalence of those aged 65 and older stands at 8%, compared to 12% in London and 18% in England as a whole, while 60% describe their ethnicity as other than white British, compared to 55% in London and 80% in England.

### Data variables and measurement

**Fig. 1:**
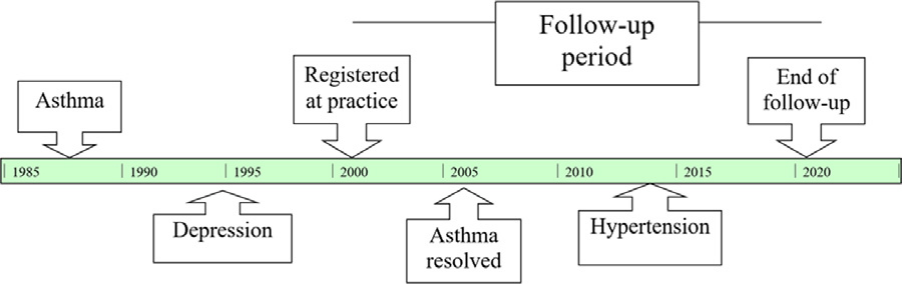
Example of a patient's trajectory as recorded in the dataset. This patient has 2 LTCs recorded onset prior to start of follow-up; 1 LTC recorded onset, and 1 LTC recorded resolved during follow-up. They ended their follow-up with the same number of LTCs as the start, and are therefore considered in a “stable” state than when they first registered for the purpose of descriptive analyses.

Dates of the start (registration or turning 18 years of age if already registered) and end (deregistration/death) of follow up are included, with end of follow-up censored at 2/5/2020 if the person was still registered. An example of a patient's trajectory and recording of condition onset and resolution is presented in Fig. 1).

Multimorbidity was defined as the co-occurrence of two or more out of 32 LTCs as reported previously.^25^^,^^26^ Conditions were selected based upon seven evaluation domains, including prevalence, impact and preventability, as well as their importance within the local urban, multi-ethnic community. Conditions and risk factors were defined using Read codes from multiple sources, including the Quality and Outcomes Framework (QOF) where applicable.^25^

We identified the first record for each of 32 long term condition as the inception date; for 12 conditions we also identified resolution or remission dates (Supplementary Fig 1) Remission refers to a clinical condition which may come and go, such as asthma, while resolve refers to conditions which recover completely, such as Hepatitis B/C. The final 12 conditions with resolve or remission dates available in the dataset included alcohol dependency, atrial fibrillation, asthma, cancer, CKD, chronic pain, depression, Type 2 diabetes, epilepsy, hypertension, morbid obesity, and substance dependency. All cancer (*n* = 2649) resolve dates occurred prior to start of follow-up and so did not contribute to the “improving” transitions observed during the follow-up period. The six risk factors considered in this study include: hypertension; moderate obesity (BMI 30·0–39·9 kg/m2), high cholesterol (total cholesterol > 5 mmol/L), smoking, high alcohol consumption (>14 units per week), and psychoactive substance use. High alcohol, moderate obesity, and smoking have resolved records during the registration period.

Sociodemographic characteristics include age, sex, and self-assigned ethnicity. Social and material deprivation was derived from participant postal code of residence were based on the Indices of Multiple Deprivation (IMD) 2019^27^ classification at lower super output area, divided into quintiles based on the national distribution. The IMD is determined based on domains including income; employment; education, skills and training; health and disability; crime; barriers to housing and services; and deprivation to the living environment.

### Statistical analysis

*Descriptive analysis:* To investigate the association between health inequality risk factors, patients were categorized depending on whether they ended their follow-up with the same number of LTCs (“Stable”), had more LTCs or died at end of follow-up (“Progressed”) or if they had less conditions at the end of follow-up than when they first started (“Remitted”). All sociodemographic characteristics and risk factors were summarized for each grouppopulation using means and standard deviations, median and inter-quartile range (IQR), or counts and percentages as appropriate. Missing data were retained as missing.

LTC accumulation from start to end of follow-up was visualized using mean cumulative function (MCF) plots. These plots define a staircase function that tracks the accumulated number of LTC occurrences over time LTCs (Supplementary Table 1), and accounts for follow-up time and death as a censoring event but disregards resolution and reduction in number of LTCs.^28^ MCFs were plotted according to number of LTCs at start of follow-up, age at start and sociodemographic and risk factor sub-groups.

**Fig. 2:**
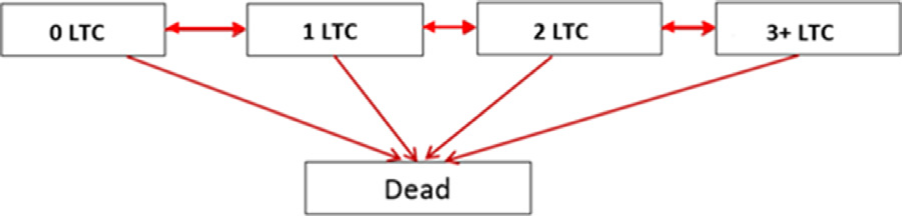
Representation of the five state Markov model. The model assumes movement up or down states, with death as the absorbing (final) state.

*Model specification:* Multistate Markov model with five states were employed to estimate probabilities of LTC progression, (Fig. 2). A Markov model is a probabilistic model that incorporates multiple states (in our case diseases and their combinations) enabling modelling of the rate of transitions between states. Five states were employed (Fig. 2) because it was sufficient to model up to three-condition states, together with states for no LTCs and death. Separate models with similar structure were fitted to each sex, ethnicity, IMD and risk factor variable, where the transition intensities are modelled as a function of these variables and changes in intensities between variable categories are presented as hazard ratios and their confidence intervals. All models were adjusted for age at start of follow-up (categorized into 18–39, 40–59, 60–79, and 80+), and the estimated probabilities of moving from one state to their state at the end of a one-year period are given for those who enter the study at age 40–59 (Supplementary Tables 3a to 3q). We assumed that people could only gain or resolve a single LTC or die at any timepoint

First order Markov chains were then applied to study the sequence of development of selected conditions (Supplementary Fig 2). Transition probabilities from these models can be interpreted as the probability of acquiring condition B after already having condition A, in one time stepWith 32 conditions, there are 31 × 31 different probabilities to assess. As it may be difficult to find interesting associations, we selected the three conditions with the highest probability in each column (antecedent conditions) and each row (consequent conditions) of the transition matrix.^20^

Both the five state and first order Markov chain models described have the key property of “memorylessness”. That is, we can predict the next state of the process only based on the current state and this prediction will be as good as the prediction based on the full history.^20^

R version 4·0·4 was used for all analyses, with the packages ‘msm’ for multistate models and “clickstream” for first order Markov chains.

### Role of the funding source

The funder had no role in the study design; in the collection, analysis, and interpretation of data; in the writing of the report; or in the decision to submit the paper for publication.

## Results

### Descriptive data

During the study period of up to 15·5 years, 826,936 patients were registered within a general practice. Five patients did not have a specified end date, and 61 had start dates equal to end dates; these were removed from analysis. 631,760 (76%)entered the study (registered, or turned 18) with no LTCs, 121,424 (15%)with 1 LTC, 41,720 (5%)with 2 LTCs, and 31,966 (4%)with 3 or more LTCs (Supplementary Table 2). There were 27,021 (3%) with a recorded date of death. In one year (1st April 2018 to 31st March 2019), patients in the most deprived quintile had a median of two GP consults (IQR = 6) versus a median of 1 (IQR = 5) in the least deprived quintile. There were 630,783 (76%) who ended their follow-up with the same number of LTCs as when they first started (Table 1); 615,565 (74%) neither gained or resolved an LTC throughout their follow-up, or died, so models of the transition rates between states in the Markov models will be largely informed by the remaining 26% who either acquired a LTC or whose LTC resolved during their follow-up period.

**Table 1:** Characteristics of patients, stratified according to their state at end of follow-up (compared to initial state). Results are given as means (standard deviations), medians (IQR) or n (row percent).

		**Overall**	**Stable**^a^	**Progressed**	**Remitted**
		826870	630783	180088	15999
Number of LTCs acquired (%)	0 LTC	632093	607998 (96)	8414 (1)	15681 (2)
	1 LTC	106825	15579 (15)	90940 (85)	306 (0)
	2+ LTCs	87952	7206 (8)	80734 (92)	12 (0)
Number of LTCs resolved (%)	0 LTC	781853	616193 (79)	165660 (21)	0 (0)
	1 LTC	43109	13918 (32)	13682 (32)	15509 (36)
	2+ LTCs	1908	672 (35)	746 (39)	490 (26)
Registration year (%)	2005 or prior	295596	198557 (67)	85890 (29)	11149 (4)
	2006–2010	173524	133156 (77)	33752 (19)	6616 (4)
	2011–2015	194538	155487 (80)	32658 (17)	6393 (3)
	2016–2020	163273	143545 (88)	16555 (10)	3173 (2)
Age at registration	18–39	617693	511097 (83)	92735 (15)	13861 (2)
	40–59	150318	96310 (64)	52157 (35)	1851 (1)
	60–79	46955	19999 (43)	26684 (57)	272 (1)
	80+	11904	3377 (28)	8512 (72)	15 (0)
Age at registration (mean (SD))	33·9 (14·7)	33·9 (14·7)	31·5 (12·3)	42·4 (18·8)	28·9 (10·9)
Age at de-registration/death (mean (SD))	39·6 (16·2)	39·6 (16·2)	36·4 (13·5)	51·2 (19·5)	35·4 (12·2)
Years registered (median (IQR))	4·2 [1·8, 8·4]	4·2 [1·8, 8·4]	3·4 [1·6, 7·0]	8·5 [4·0, 15·1]	5·3 [2·7, 9·3]
Sex (%)	Male	396431	306346 (77)	82399 (21)	7686 (2)
	Female	430434	324433 (75)	97688 (23)	8313 (2)
Ethnicity (%)	White	445865	338692 (76)	97295 (22)	9878 (2)
	Black	113775	72205 (63)	39544 (35)	2026 (2)
	Asian	49941	37679 (75)	11436 (23)	826 (2)
	Mixed	31236	23129 (74)	7366 (24)	741 (2)
	Other	23745	18944 (80)	4538 (19)	263 (1)
	Missing	162308	140134 (86)	19909 (12)	2265 (1)
IMD national quintile (%)	1 - most deprived	144107	104692 (73)	36941 (26)	2474 (2)
	2	386623	296211 (77)	83133 (22)	7279 (2)
	3	218040	168381 (77)	45156 (21)	4503 (2)
	4	56689	44515 (79)	10947 (19)	1227 (2)
	5 - least deprived	11390	9076 (80)	1960 (17)	354 (3)
	Missing	10021	7908 (79)	1951 (19)	162 (2)
Alcohol 14+ units(%)	Never	815605	623178 (76)	176768 (22)	15659 (2)
	Ever had^b^	9978	6971 (70)	2695 (27)	312 (3)
	Resolved^c^	1287	634 (49)	625 (49)	28 (2)
Total cholesterol > 5 mmol/L (%)	Never	673228	558318 (83)	101152 (15)	13758 (2)
	Ever had^b^	153642	72465 (47)	78936 (51)	2241 (1)
Moderately obese (%)	Never	717879	573418 (80)	130530 (18)	13931 (2)
	Ever had^b^	93057	51509 (55)	39715 (43)	1833 (2)
	Resolved^c^	15934	5856 (37)	9843 (62)	235 (1)
Smoking	Never	479935	389367 (81)	81939 (17)	8629 (2)
	Ever had^b^	211882	161229 (76)	46635 (22)	4018 (2)
	Resolved^c^	135053	80187 (59)	51514 (38)	3352 (2)
Substance use	Never	802710	618317 (77)	168838 (21)	15555 (2)
	Ever had^b^	24160	12466 (52)	11250 (47)	444 (2)

a Stable = Same number of LTCs than at start of registration; Progressed = More LTCs than at start of registration, or died; Remitted = Less LTCs than at start of registration.

b Any record of the risk factor either prior to or during the registration period, but never resolved.

c Resolved during period of registration IMD = Indices of Multiple Deprivation; LTCs = Long term conditions.

Patients were categorized depending on whether they ended their follow-up with the same number of LTCs (“Stable”, 630,783), had more LTCs or died at end of follow-up (“Progressed”, 180,088) or if they had less conditions at the end of follow-up than when they first started (“Remitted”, 15,999; Table 1). Those who remitted had one or more LTCs resolved, and most (*n* = 15,681) had no LTCs gained during their follow-up period. Those who progressed had at least 1 or more LTCs gained, and very few had LTCs resolved during their follow-up period.

Those who have progressed were older (mean age of 42·4 years at registration) and had been registered at the practice for longer (median follow-up period of 8·5 years) compared to those who were stable or remitted (Table 1). There were 35% of Black ethnic groups who progressed compared 22% for White ethnic groups, while 26% in the most deprived group progressed compared to 17% in the least deprived group. A higher proportion of patients who have resolved risk factors progressed (for example, 49% (*n* = 625) of those with resolved alcohol progressed vs 27% (*n* = 2695) who have a record of high alcohol consumption).

**Fig. 3:**
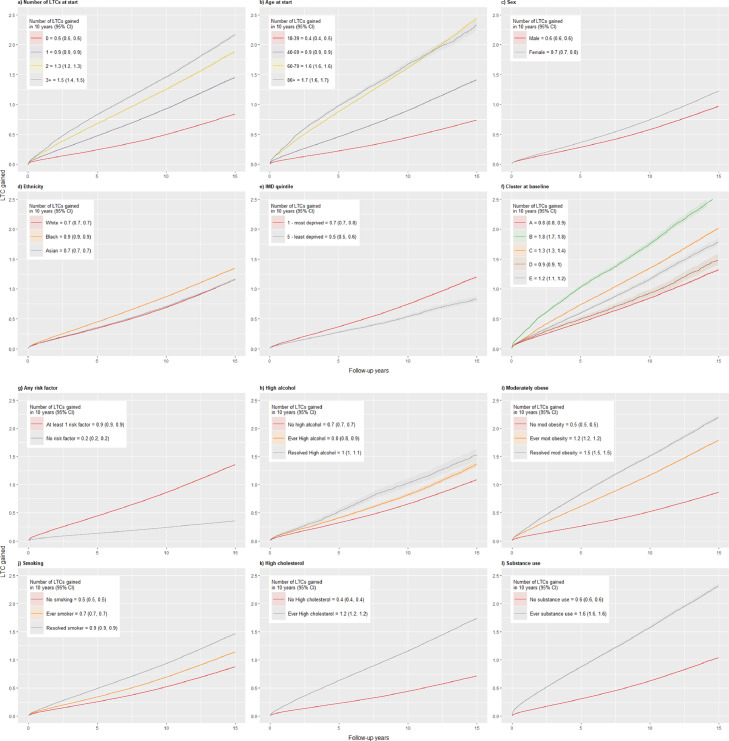
a – 3l: Mean cumulative function plots showing the average time it takes to acquire LTCs, in years since start of follow-up “Ever” risk factor = Any record of the risk factor either prior to or during the registration period, but never resolved “Resolved” risk factor = Resolved during period of registration “No” risk factor = No record of the risk factor at any point ^a^Results from cluster analysis from Bisquera et al.1 A) anxiety and depression (the “mental health” cluster); B) heart failure, atrial fibrillation, CKD, CHD, stroke/TIA, PAD, dementia, and osteoporosis (the “cardiovascular” cluster); C) osteoarthritis, cancer, chronic pain, hypertension, and diabetes (the “pain” cluster); D) chronic liver disease and viral hepatitis (the “liver disease” cluster); E) substance and alcohol dependency and HIV (the “dependence” cluster); F) conditions not identified as highly correlated with any particular cluster.

The mean number of LTCs gained over the 15·5-year follow up were plotted in a mean cumulative function plot for each subgroup (Fig. 3a-3l). These plots measure the accumulation of LTCs and do not account for resolved conditions. The rate of LTC accumulation increases according to baseline number of LTCs, age, female sex, Black ethnic groups, deprivation, and having risk factors. Cluster B have the fastest rate of accumulation, gaining an additional 1.7 to 1.8 conditions on average within a span of 10 years. Similar to the results in Table 3, those with “resolved” risk factors have a faster rate of accumulation of LTCs compared to those who have “ever had” a risk factor but not resolved.

### LTC progression

Table 2 compares the transition intensities of different subgroups moving to each state (which includes death, multimorbidity progression and resolution), in the form of hazard ratios (HRs). Females, compared to males, are more likely to move up and down states (gain or lose LTCs) with HRs over 1, however males are more likely to die regardless of how many LTCs they have. People from Black ethnicity compared to White ethnicity are more likely to move between states of multimorbidity. They are more likely to die without acquiring any conditions (0 LTC -> Death; HR: 1 0·08, 1·77), but are less likely to die with multimorbidity (2 -> Death; HR: 0·62, 0·90)). Similar patterns can be seen with people with Asian ethnicity compared to White ethnicity, with increased hazards when moving between 0->1 and 1->2 LTC states but decreased hazards when moving between 2->3 LTC or to death. No differences in hazards are seen in the remitting transitions (1->0 and 3+ ->2LTC) between the Asian and White ethnicity groups. People from deprived areas are more likely to acquire LTCs and are more likely to die with multimorbidity compared to those in affluent areas (2 LTC -> Death; HR: 1·29, 12·80). Having at least 1 risk factor increases the likelihood of gaining conditions, however appears to decrease the likelihood of death. In particular, high alcohol consumption (ever), moderate obesity (ever and resolved), smoking (ever and resolved), and substance use increases the likelihood of transition from 1- > 2LTC and 2 -> 3+LTC. Moderate obesity (ever), smoking (resolved), and high cholesterol (ever) are associated with lower hazards of death with conditions (1/2/3+ LTC -> Death). High alcohol consumption is not associated with changing hazards of death from any number of conditions. Changing the groupings of patients in different obesity categories did not make an appreciable difference to the estimates (Supplementary Table 5).

**Table 2:** Hazard ratios of moving to each state, with 95% confidence intervals. All estimates are adjusted to age at start of follow-up*.

	Progressing transitions							Resolving/Remitting transitions		
	0 -> 1 LTC	1 -> 2 LTC	2 -> 3+ LTC	0 -> Death	1 -> Death	2 -> Death	3+ -> Death	1-> 0 LTC	2 -> 1 LTC	3+ -> 2 LTC
Age 40–59 vs 18–39	1·68 (1·66, 1·71)	1·87 (1·81, 1·94)	1·33 (1·29, 1·36)	3·00 (2·44, 3·69)	4·17 (3·49, 4·98)	3·22 (2·62, 3·95)	2·95 (2·71, 3·22)	0·44 (0·42, 0·46)	1·31 (1·22, 1·39)	0·38 (0·36, 0·40)
Age 60–79 vs 18–39	3·63 (3·53, 3·73)	7·88 (6·72, 9·24)	2·22 (2·15, 2·3)	36·72 (30·65, 43·98)	16·53 (13·55, 20·18)	9·70 (7·86, 11·97)	10·22 (9·42, 11·09)	0·39 (0·34, 0·44)	4·90 (4·04, 5·94)	0·19 (0·17, 0·21)
Age 80+ vs 18–39	4·04 (3·79, 4·31)	NA	4·49 (4·23, 4·77)	NA	NA	NA	33·77 (31·05, 36·72)	0·28 (0·18, 0·42)	NA	0·27 (0·22, 0·33)
Female vs Male	1·50 (1·48, 1·52)	1·17 (1·13, 1·20)	1·02 (1·00, 1·05)	0·75 (0·66, 0·85)	0·43 (0·37, 0·50)	0·65 (0·58, 0·72)	0·67 (0·65, 0·69)	1·05 (1·02, 1·08)	1·02 (0·98, 1·07)	0·87 (0·83, 0·92)
Black vs White	1·36 (1·33, 1·39)	1·25 (1·20, 1·30)	0·98 (0·96, 1·01)	1·38 (1·08, 1·77)	0·69 (0·54, 0·88)	0·74 (0·62, 0·90)	0·67 (0·65, 0·70)	0·91 (0·87, 0·95)	1·20 (1·13, 1·28)	0·98 (0·92, 1·04)
Asian vs While	1·03 (1·00, 1·06)	1·2 (1·13, 1·28)	0·92 (0·88, 0·96)	0·88 (0·59, 1·32)	0·62 (0·41, 0·95)	0·66 (0·48, 0·91)	0·71 (0·66, 0·75)	1·04 (0·98, 1·11)	1·22 (1·09, 1·36)	0·98 (0·88, 1·10)
IMD most vs least deprived	1·06 (0·99, 1·13)	1·46 (1·30, 1·64)	1·16 (1·03, 1·30)	2·24 (1·01, 4·98)	2·31 (1·03, 5·17)	4·06 (1·29, 12·80)	1·59 (1·32, 1·91)	0·68 (0·60, 0·76)	1·23 (1·01, 1·49)	0·69 (0·54, 0·89)
At least one risk factor vs none	2·77 (2·72, 2·81)	1·69 (1·63, 1·74)	1·47 (1·40, 1·53)	0·40 (0·34, 0·47)	0·93 (0·74, 1·17)	0·15 (0·13, 0·17)	0·49 (0·45, 0·53)	0·97 (0·94, 1·00)	0·95 (0·9, 1·00)	0·49 (0·45, 0·53)
Ever alcohol vs none	1·61 (1·50, 1·72)	1·43 (1·23, 1·68)	1·00 (0·92, 1·09)	0·23 (0·04, 1·36)	0·35 (0·11, 1·06)	1·18 (0·72, 1·94)	1·06 (0·93, 1·22)	1·68 (1·50, 1·89)	1·85 (1·50, 2·28)	0·88 (0·74, 1·05)
Resolved alcohol vs none	1·53 (1·23, 1·90)	2·00 (0·67, 5·98)	0·87 (0·72, 1·06)	NA	NA	NA	NA	1·40 (0·92, 2·13)	2·57 (0·76, 8·68)	0·80 (0·57, 1·12)
Ever moderate obese vs none	2·95 (2·88, 3·03)	1·75 (1·69, 1·81)	1·29 (1·26, 1·32)	0·20 (0·11, 0·37)	0·29 (0·24, 0·36)	0·36 (0·28, 0·45)	0·58 (0·56, 0·61)	1·61 (1·53, 1·69)	1·4 (1·33, 1·48)	0·78 (0·74, 0·83)
Resolved moderate obese vs none	NA	1·16 (1·09, 1·23)	1·34 (1·29, 1·39)	NA	0·12 (0·07, 0·21)	0·01 (0, 1·08)	0·6 (0·57, 0·63)	NA	0·74 (0·67, 0·82)	0·66 (0·59, 0·73)
Ever smoker vs none	1·29 (1·27, 1·32)	1·40 (1·35, 1·44)	1·3 (1·27, 1·34)	0·97 (0·81, 1·15)	3·02 (2·58, 3·53)	1·6 (1·42, 1·81)	1·8 (1·74, 1·87)	0·89 (0·86, 0·93)	1·27 (1·20, 1·34)	0·80 (0·75, 0·85)
Resolved smoker vs none	2·12 (2·07, 2·16)	1·22 (1·17, 1·26)	1·21 (1·18, 1·24)	0·07 (0·03, 0·16)	0·74 (0·61, 0·90)	0·34 (0·29, 0·41)	1·00 (0·97, 1·04)	1·34 (1·28, 1·39)	1·02 (0·97, 1·08)	0·85 (0·80, 0·90)
Ever cholesterol vs none	NA	0·81 (0·79, 0·84)	1·09 (1·07, 1·12)	1·53 (1·30, 1·80)	NA	0·20 (0·17, 0·24)	0·55 (0·54, 0·57)	NA	0·70 (0·66, 0·73)	0·59 (0·55, 0·62)
Ever substance use vs none*	NA	NA	1·06 (1·02, 1·09)	NA	NA	NA	0·55 (0·52, 0·58)	NA	NA	0·55 (0·51, 0·61)

⁎ Substance use is unable to be age adjusted due to low counts “Ever” risk factor = Any record of the risk factor either prior to or during the registration period, but never resolved “Resolved” risk factor = Resolved during period of registration “None” risk factor = No record of the risk factor at any point “NA” = Confidence intervals from the Hazard ratios were unable to be calculated from the model, due to low numbers in these transitions IMD = Indices of Multiple Deprivation; LTCs = Long term conditions.

Individual predicted probabilities of moving across states over a one- year period, along with the mean sojourn time at each state are given in Supplementary Tables 3a to 3q; a selection of these probabilities are summarized in Table 3. The probabilities are very high of gaining LTCs over a one- year period if they have 1 or more risk factors, particularly for substance use where there is an 85% chance of gaining LTCs within the next year, and where people stay less than a year at zero LTCs on average before acquiring conditions.

**Table 3:** Selected^a^ probabilities from the transition matrices of each multi-state model, for people who enter the study at age 40–59^b^.

	Annual probability of transition to higher morbidity or death, if currently no LTC	Annual probability of transition to higher morbidity or death, if currently multimorbid
Male	0·06 (1 in 17)	0·12 (1 in 8)
Female	0·09 (1 in 11)	0·12 (1 in 8)
IMD1 - most deprived	0·08 (1 in 13)	0·14 (1 in 7)
IMD5 - least deprived	0·07 (1 in 14)	0·12 (1 in 8)
White	0·09 (1 in 11)	0·11 (1 in 9)
Black	0·11 (1 in 9)	0·13 (1 in 8)
Asian	0·09 (1 in 11)	0·13 (1 in 8)
At least 1 risk factor	0·09 (1 in 11)	0·12 (1 in 8)
No risk factors	0·04 (1 in 25)	0·14 (1 in 7)
Alcohol ever	0·11 (1 in 9)	0·21 (1 in 5)
Moderate obesity ever	0·15 (1 in 7)	0·14 (1 in 7)
Smoking ever	0·07 (1 in 14)	0·14 (1 in 7)
High cholesterol ever	0·68 (1)	0·08 (1 in 12)
Substance use ever	0·85 (1)	0·07 (1 in 14)

a All estimated probabilities of moving from one state to the next can be found in Supplementary Tables 3a to 3q.

b Substance use is unable to be age adjusted due to low counts “Ever” risk factor = Any record of the risk factor either prior to or during the registration period, but never resolved “None” risk factor = No record of the risk factor at any point IMD = Indices of Multiple Deprivation; LTCs = Long term conditions.

### Probabilities of follow-up conditions

Supplementary Table 4a – 4j presents the results of the first order Markov chain models, whereby the top 3 antecedents and consequents are given for each condition and death (more than 3 if there are equal probabilities, or less than 3 if the probabilities are zero), along with cluster membership and prevalence. The results are consistent with the clusters results,^26^ with each condition frequently being preceded or succeeded by another condition within the same cluster. For example, substance and alcohol dependency go hand in hand and cardiovascular conditions are often found together. Anxiety is the most prevalent condition for both sex, the White ethnicity population, and least deprived groups. This condition is often preceded by depression, asthma and IBD, and succeeded by depression, hypertension, and chronic pain. In the Black ethnicity population and those in the most deprived groups, chronic pain and hypertension are the most prevalent. These are often preceded by type 2 diabetes, lupus, morbid obesity, sickle cell disease and osteoarthritis, and are succeeded by type 2 diabetes, depression, anxiety, and osteoarthritis.

## Discussion

### Key findings

In this study, we present differences in multiple LTC trajectory patterns in terms of a) the probabilities of acquiring and resolving/remitting conditions over time and b) the probabilities of each condition being preceded and succeeded by other conditions. We found that certain groups (females, Black and Asian ethnicity people) are more likely to be more dynamic with frequent movement up and down states, whereas males and White ethnic groups are more likely to stay at a particular state or die with multimorbidity. Those in the most deprived quintile are more likely to gain LTCs or die with multimorbidity, and less likely to resolve their conditions. Smoking (ever) increases the hazards of death, particularly if a person already has one or more conditions, but the risk factors high cholesterol and moderate obesity (ever and resolved) appear to have a protective effect with reduced hazards of death when compared to those without these risk factors. Patients with a record of substance use have an 85% chance of gaining LTCs over a one-year period, and they stay less than a year at a healthy state on average before acquiring conditions.

The differences seen between ethnicities and the unexpected finding with the resolution of risk factors may be attributed to differences in data monitoring over time. People from White backgrounds may stay within the borough, have more accurate death and LTC records and are followed up for longer, hence why it may appear that they have a higher mortality rate and are more likely to progress to a severe (3+ LTC) state of multimorbidity. This may also explain why people with a record of resolved risk factors end up with more LTCs over time – this could suggest that a) these people are attempting to control their lifestyle risk factors once they have been diagnosed with increasing morbidities or b) they have more risk factor monitoring which is likely to increase prevalence

Examination of individual LTCs show results that are consistent with the clusters identified in a previous cluster study,^26^ with each condition likely to be preceded or succeeded by another condition within the same cluster. The mental health conditions are also linked strongly to the highly prevalent physical conditions hypertension and chronic pain. In people from White ethnic backgrounds conditions such as anxiety, depression, hypertension, chronic pain asthma and IBD are linked, whereas in Black ethnicity people we see associations between type 2 diabetes, lupus, morbid obesity, and osteoarthritis.

### Comparison with existing literature

There is already a strong body of evidence about the relationship between deprivation and multimorbidity in two respects: higher prevalence, earlier age of onset.^6^ This study adds a third dimension, providing evidence that progression of MM is more severe (‘accelerated development’ or higher LTC acquisition rate) in deprived areas.

Studies examining multimorbidity trajectories are scarce, however ours are in line with previous results in that the most likely transition is to continue with the same number of conditions or conditions from the same cluster.^10^ Our study also indicates that a higher number of LTCs at baseline relates to a faster rate of accumulation – those with zero LTCs at the start take over 15 years on average to gain a condition, compared to 6.4 years for those with 3 or more conditions. Advanced age was associated with increased likelihood of development of multimorbidity in those with no conditions at baseline, but also a faster rate of accumulation in those who already have one or more conditions.^4^^,^^29^ Changes in multimorbidity were also found to be a greater predictor of mortality than baseline multimorbidity in adults, and that earlier multimorbidity onset results in greater life-year-lost.^30^^,^^31^

Outside of age, poor socioeconomic status^32^^,^^33^ and unhealthy behaviours, particularly alcohol consumption, obesity and smoking^17^^,^^29^^,^^34^ are the key determinants of a worsening multimorbidity trajectory. Evidence from this study and previous work using the same dataset^24^ suggests that those in the most deprived quintile can gain an additional LTC up to 3 years earlier than those in the least deprived (Fig. 3) and that risk factors, particularly substance use, may be the biggest determinant for multimorbidity development in a younger population.

Two studies also found differences in multimorbidity trajectory patters by ethnicity. Compared to White participants, Black American participants had a higher rate of multimorbidity at baseline along with a slow rate of disease accumulation over time while Hispanic participants tended to start their trajectory with fewer conditions but to acquire conditions at a faster rate.^11^ White individuals were more likely to transition from disease to death,^21^ which is consistent with our study.

All studies we have encountered on multimorbidity acquisition assume trajectories move towards progression (gaining LTCS, or death) only. While our results reflect impacts of the social inequalities identified in previous studies – i.e. that females and the Black ethnicity population have more disadvantages in regards to their mLTC journey^6^^,^^24^ – the full picture is more nuanced. These groups are more likely to be in a more fluctuating state of change by gaining and losing LTCs, but are less likely to die with them. The increased prevalence of the most common conditions –depression in females and chronic pain in the Black ethnicity population may explain these findings. These conditions are likely to be regularly monitored hence the increased identification and recording of resolved.

Our results agree with previous studies, with relationships detected between the common mental and physical conditions including chronic pain and IBD,^35^ between cardiovascular conditions and dementia,^36^ between alcohol dependency, substance dependency and HIV, cancer and cardiometabolic diseases,^23^ and with diabetes as a precursor to hypertension and chronic pain.^20^^,^^22^

### Strengths and limitations

To our knowledge, this study is the first to examine both LTC acquisition and resolution over time and the associations among 32 conditions. While one study used Markov chains to give the probabilities of relationships among 103 conditions,^20^ and another study used associated rule mining to identify strong associations among LTCs using the UK biobank data,^37^ neither took disease sequences into account. Detailed studies on disease transitions partially depict the process of LTC accumulation by focusing on a limited number of diseases,^10^^,^^21^ which hinders its usefulness for policy strategies. The methods used in this study could be applied and validated on larger datasets to give personalised information, both to patients and clinicians, on the likelihood of acquiring specific conditions and expected disease patterns.

Data extracted from electronic health records, particularly in primary care, are known for under-counting conditions.^38^ Thus, prevalence estimates of LTCs reported in this study are likely to be an under-estimate. This study is limited to specific LTCs and risk factors and did not investigate other health complaints such as frailty, acute conditions and surgical conditions. Conversely, the summary of the total number of LTCs depicted in the mean cumulative incidence estimates might be slightly biased upwards due to the potential competing risk issue from death. The conditions in our study can be identified as resolved/remiss based on a standardised ruleset from the QOF (e.g. depression), but for non-QOF conditions (e.g. anxiety) ‘resolve’ codes are available but were not applied consistently. This means the relationship between anxiety and depression may change had coding of resolved conditions been more consistent. Relating to this, it is difficult to disentangle true population changes over time from increased data recording over time. Changes in LTC prevalence may be attributable to improved data recording, or true population changes.

In addition, there were concerns over the accuracy of death records within this dataset. Despite what was initially thought of as a low death rate (3% across the entire population), after age standardization, death rates appear to be higher prior to 2013, when compared to national records (Supplementary Fig 3). Possible reasons include improvements to data accuracy over time, or to ‘list cleansing’, whereby NHS England automatically de-registered all patients who had not seen their GP within three years (with a six-month period of grace, during which the GP could appeal and request reinstatement).^9^

A limitation of our analysis is that due to the large number of conditions, a first-order Markov chain was assumed. The memoryless property of this model meant that the full sequence of past events could not be considered. Using a more complicated model (i.e., a higher order Markov chain) where sequences are examined further back in time would result in a vast and highly uninterpretable output. This is also the reason why we limited our multi-state analysis to five states (with the highest state being three or more LTCs), as opposed to examining the acquisition of four, five and more LTCs individually.

## Conclusion

We examined the relations among 32 conditions, taking the order of disease occurrence and resolution into consideration. Distinctive patterns for the development and accumulation of multimorbidity have emerged, with confirmation of disadvantages seen in least vs most deprived quintile, and in relationships seen between specific conditions. People from Black and Asian ethnicity, females, and unhealthy risk factors were associated with continuous transitions between multimorbid states, and less time spent in a healthy state (0 LTC), when compared to people of White ethnicity and males. Future research in multimorbidity needs to recognize that adults develop multimorbidity at different rates over time. The methods detailed in this paper can be applied to larger datasets to derive probabilities of developing multimorbidity within specific groups to inform clinical practice and interventions to improve health outcomes for people with multimorbidity.

## Author's contributions

**Alessandra Bisquera:** Conceptualization, Methodology, Software, Validation, Formal analysis, Writing - Original Draft, Writing - Review & Editing, Investigation, Visualisation, Project administration; **Ellie Bragan Turner**: Writing - Review & Editing, Validation; **Rupert Dunbar-Rees**: Writing - Review & Editing, Validation; **Nasrin Hafezparast**: Writing - Review & Editing, Validation; **Martin Gulliford:** Writing - Original Draft, Writing - Review & Editing, Funding acquisition; **Hiten Dodhia**: Project administration, Conceptualization, Resources, Writing - Review & Editing, Investigation, Funding acquisition; **Lesedi Ledwaba-Chapman:** Validation, Formal analysis, Writing - Review & Editing; **Stevo Durbaba:** Data curation, Software; **Marina Soley-Bori:** Writing - Review & Editing; **Julia Fox-Rushby:** Writing - Review & Editing, Funding acquisition; **Mark Ashworth:** Conceptualization, Methodology, Resources, Writing - Review & Editing, Investigation, Supervision, Project administration, Funding acquisition; **Yanzhong Wang:** Conceptualization, Methodology, Resources, Writing - Review & Editing, Investigation, Supervision, Funding acquisition. All authors have verified the underlying data and accept responsibility to submit for publication.

## Data sharing

The data are not publicly available to share, but the research group can provide descriptive data in table form. Requests should be made to Mark Ashworth (mark.ashworth@kcl.ac.uk).

## Patient consent for publication

Not required.

## Ethics approval

All data were extracted under the terms of a signed data sharing agreement with each practice and with project-specific approval following submission of a data privacy impact assessment, approved by Lambeth Clinical Commissioning Group in 2 November 2017. Information governance approval required ‘low number suppression’, ensuring that data could not be displayed if the patient number was 10 or less in any given category; in these circumstances, data reporting would state: ‘≤10 patients’. Separate ethical committee approval was not required (Health Research Authority, personal correspondence, 29 September 2017) since all data were fully anonymised for the purposes of research access, and all patient identifiable data had been removed.

## Funding

The study was supported by a grant from Impact on Urban Health, London, United Kingdom (Charity No: 1160316).

## Declaration of interests

The authors declare no conflict of interest.

## Appendix Supplementary materials

Image, application 1
