# Supplementary material for: Inequalities in developing multimorbidity over time: A population-based cohort study from an urban, multi-ethnic borough in the United Kingdom
Source: Lancet Reg Health Eur. 2021 Nov 4;12:100247. doi: 10.1016/j.lanepe.2021.100247 (PMC8640725; doi:10.1016/j.lanepe.2021.100247)
Supplement: Supplementary file 1 [file mmc1.docx]

Supplementary Tables and Figures

Supplementary Table 1 Example data for one patient, showing time since registration (in years), total number of LTCs, and "State" analysed in the five state (indicated by 1 to 5) Markov model

| **ID** | **Time** | **Age** | **Total LTC** | **Death** | **State** |
| --- | --- | --- | --- | --- | --- |
| 117 | 0.00 | 69.00 | 2 | 0 | 3 |
| 117 | 0.12 | 69.12 | 3 | 0 | 4 |
| 117 | 0.41 | 69.41 | 4 | 0 | 4 |
| 117 | 5.25 | 74.25 | 5 | 0 | 4 |
| 117 | 5.35 | 74.35 | 6 | 0 | 4 |
| 117 | 5.54 | 74.54 | 6 | 1 | 5 |

Supplementary Table 2 Observed transitions from the start to the end of registration period (n=826,870). Results are given as n (column percent)

|  | Start of registration | | | |
| --- | --- | --- | --- | --- |
|  | 0 LTC | 1 LTC | 2 LTC | 3+ LTC |
| **End of registration** | 631760 (76%) | 121424 (15%) | 41720 (5%) | 31966 (4%) |
| 0 LTC | 522154 (82.7) | 12451 (10.3) | 85 (0.2) | 4 (0.0) |
| 1 LTC | 64507 (10.2) | 68722 (56.6) | 2499 (6.0) | 72 (0.2) |
| 2 LTC | 24084 (3.8) | 18055 (14.9) | 18964 (45.5) | 888 (2.8) |
| 3+ LTC | 15127 (2.4) | 16336 (13.5) | 14958 (35.9) | 20943 (65.5) |
| Dead | 5888 (0.9) | 5860 (4.8) | 5214 (12.5) | 10059 (31.5) |

**Supplementary Table 3a to 3q:**

Individual predicted probabilities of moving across states over a ten-year period, along with the mean sojourn time at each state are given in Supplementary tables 1a to 1m. These probabilities can be interpreted as follows: those Females aged 40-59 who currently have no LTCs have a 52% chance remaining at no LTCs, 27% chance of acquiring 1, 15% chance of acquiring 2, 14% chance of acquiring 3 or more, and a 2% chance of death within the next 10 years. On average these people are likely to stay at a healthy state (0 LTC) for 9.3 years before moving to another state.

When comparing probabilities, we find that females are less likely to stay at a healthy state (0 LTC) compared to males, however, are less likely to die after acquiring LTCs. The same goes for Black and Asian ethnic groups compared to White – Black ethnicity people stay at 0 LTC for 8.1 years on average compared to 11 years for White ethnicity people. Those in the most deprived quintile are more likely to remain multimorbid (2 LTC) or severely multimorbid (3+ LTC), with a length of stay of 24.3 years compared to 22.2 years in the least deprived quintile. For those who currently have no LTCs, the probability of acquiring 1 or more LTCs over the next 10 years is 58% if they have a record of at least one risk factor; this goes to over 90% if they have a record of substance use. If they have no risk factors, then they have a 73% chance of remaining at 0 LTC over the next 10 years.

Supplementary Table 3a to 3q Estimated probabilities of moving from one state to their state at the end of a one year period, for people who enter the study at age 40-59

| **Female** | Next State | | | | | Mean sojourn time in years (95% CI) |
| --- | --- | --- | --- | --- | --- | --- |
| Current state | LTC 0 | LTC 1 | LTC 2 | LTC 3+ | Death |  |
| LTC 0 | 0.91 | 0.08 | 0.01 | 0.00 | 0.00 | 10.5 (10.4,10.7) |
| LTC 1 | 0.03 | 0.79 | 0.17 | 0.02 | 0.00 | 3.9 (3.0.8,4) |
| LTC 2 | 0.00 | 0.12 | 0.73 | 0.14 | 0.00 | 3.0 (2.9,3.1) |
| LTC 3+ | 0.00 | 0.00 | 0.02 | 0.97 | 0.01 | 27.5 (26.6, 28.6) |

| **Male** | Next State | | | | | Mean sojourn time in years (95% CI) |
| --- | --- | --- | --- | --- | --- | --- |
| Current state | LTC 0 | LTC 1 | LTC 2 | LTC 3+ | Death |  |
| LTC 0 | 0.94 | 0.05 | 0.00 | 0.00 | 0.00 | 15.7 (15.5,15.9) |
| LTC 1 | 0.03 | 0.81 | 0.15 | 0.01 | 0.00 | 4.4 (4.3,4.5) |
| LTC 2 | 0.00 | 0.12 | 0.73 | 0.13 | 0.01 | 3.0 (3.0,3.1) |
| LTC 3+ | 0.00 | 0.00 | 0.02 | 0.96 | 0.02 | 21.7 (20.9,22.4) |

| **White** | Next State | | | | | Mean sojourn time in years (95% CI) |
| --- | --- | --- | --- | --- | --- | --- |
| Current state | LTC 0 | LTC 1 | LTC 2 | LTC 3+ | Death |  |
| LTC 0 | 0.91 | 0.08 | 0.01 | 0.00 | 0.00 | 11.0 (10.8,11.2) |
| LTC 1 | 0.03 | 0.8 | 0.15 | 0.01 | 0.00 | 4.3 (4.2,4.4) |
| LTC 2 | 0.00 | 0.11 | 0.74 | 0.14 | 0.00 | 3.2 (3.1,3.3) |
| LTC 3+ | 0.00 | 0.00 | 0.02 | 0.96 | 0.02 | 24.3 (23.5,25.1) |

| **Black** | Next State | | | | | Mean sojourn time in years (95% CI) |
| --- | --- | --- | --- | --- | --- | --- |
| Current state | LTC 0 | LTC 1 | LTC 2 | LTC 3+ | Death |  |
| LTC 0 | 0.89 | 0.10 | 0.01 | 0.00 | 0.00 | 8.1 (7.9,8.3) |
| LTC 1 | 0.03 | 0.77 | 0.18 | 0.02 | 0.00 | 3.6 (3.5,3.7) |
| LTC 2 | 0.00 | 0.13 | 0.73 | 0.14 | 0.00 | 2.9 (2.8,3.0) |
| LTC 3+ | 0.00 | 0.00 | 0.02 | 0.97 | 0.01 | 28.4 (27.1,29.7) |

| **Asian** | Next State | | | | | Mean sojourn time in years (95% CI) |
| --- | --- | --- | --- | --- | --- | --- |
| Current state | LTC 0 | LTC 1 | LTC 2 | LTC 3+ | Death |  |
| LTC 0 | 0.91 | 0.08 | 0.01 | 0.00 | 0.00 | 10.7 (10.4,11.1) |
| LTC 1 | 0.03 | 0.78 | 0.17 | 0.01 | 0.00 | 3.7 (3.5,3.9) |
| LTC 2 | 0.00 | 0.13 | 0.73 | 0.13 | 0.00 | 3.0 (2.8,3.2) |
| LTC 3+ | 0.00 | 0.00 | 0.02 | 0.97 | 0.01 | 27.9 (25.8,30.2) |

| **IMD 1 – most deprived** | Next State | | | | | Mean sojourn time in years (95% CI) |
| --- | --- | --- | --- | --- | --- | --- |
| Current state | LTC 0 | LTC 1 | LTC 2 | LTC 3+ | Death |  |
| LTC 0 | 0.92 | 0.07 | 0.01 | 0.00 | 0.00 | 12.6 (12.4,12.9) |
| LTC 1 | 0.02 | 0.78 | 0.18 | 0.02 | 0.00 | 3.6 (3.5,3.7) |
| LTC 2 | 0.00 | 0.14 | 0.71 | 0.14 | 0.01 | 2.7 (2.6,2.8) |
| LTC 3+ | 0.00 | 0.00 | 0.02 | 0.96 | 0.02 | 24.3 (23.3,25.3) |

| **IMD 5 – least deprived** | Next State | | | | | Mean sojourn time in years (95% CI) |
| --- | --- | --- | --- | --- | --- | --- |
| Current state | LTC 0 | LTC 1 | LTC 2 | LTC 3+ | Death |  |
| LTC 0 | 0.93 | 0.06 | 0.01 | 0.00 | 0.00 | 13.5 (12.6,14.3) |
| LTC 1 | 0.03 | 0.82 | 0.13 | 0.01 | 0.00 | 4.8 (4.3,5.2) |
| LTC 2 | 0.00 | 0.12 | 0.75 | 0.13 | 0.00 | 3.3 (2.9,3.6) |
| LTC 3+ | 0.00 | 0.00 | 0.03 | 0.96 | 0.01 | 22.2 (18.3,27.0) |

| **No risk factors - Ever** | Next State | | | | | Mean sojourn time in years (95% CI) |
| --- | --- | --- | --- | --- | --- | --- |
| Current state | LTC 0 | LTC 1 | LTC 2 | LTC 3+ | Death |  |
| LTC 0 | 0.96 | 0.03 | 0.00 | 0.00 | 0.00 | 27.2 (26.7,27.7) |
| LTC 1 | 0.03 | 0.85 | 0.10 | 0.01 | 0.00 | 5.9 (5.7,6.1) |
| LTC 2 | 0.00 | 0.14 | 0.74 | 0.09 | 0.02 | 3.2 (3.1,3.4) |
| LTC 3+ | 0.00 | 0.00 | 0.04 | 0.92 | 0.03 | 12.1 (11.3,12.9) |

| **At least 1 risk factor** | Next State | | | | | Mean sojourn time in years (95% CI) |
| --- | --- | --- | --- | --- | --- | --- |
| Current state | LTC 0 | LTC 1 | LTC 2 | LTC 3+ | Death |  |
| LTC 0 | 0.91 | 0.08 | 0.01 | 0.00 | 0.00 | 10 (9.9,10.2) |
| LTC 1 | 0.03 | 0.79 | 0.16 | 0.01 | 0.00 | 3.9 (3.8,4.0) |
| LTC 2 | 0.00 | 0.12 | 0.73 | 0.14 | 0.00 | 3 (2.9,3.1) |
| LTC 3+ | 0.00 | 0.00 | 0.02 | 0.96 | 0.02 | 24.8 (24,25.6) |

| **High Alcohol - Ever** | Next State | | | | | Mean sojourn time in years (95% CI) |
| --- | --- | --- | --- | --- | --- | --- |
| Current state | LTC 0 | LTC 1 | LTC 2 | LTC 3+ | Death |  |
| LTC 0 | 0.89 | 0.10 | 0.01 | 0.00 | 0.00 | 8.4 (7.9,9.0) |
| LTC 1 | 0.04 | 0.74 | 0.20 | 0.02 | 0.00 | 2.9 (2.5,3.3) |
| LTC 2 | 0.01 | 0.20 | 0.66 | 0.13 | 0.01 | 2.1 (1.9,2.5) |
| LTC 3+ | 0.00 | 0.000 | 0.02 | 0.96 | 0.02 | 25.8 (23.0,29.0) |

| **High Alcohol - resolved** | Next State | | | | | Mean sojourn time in years (95% CI) |
| --- | --- | --- | --- | --- | --- | --- |
| Current state | LTC 0 | LTC 1 | LTC 2 | LTC 3+ | Death |  |
| LTC 0 | 0.90 | 0.09 | 0.02 | 0.00 | 0.00 | 8.9 (7.2,11.0) |
| LTC 1 | 0.03 | 0.69 | 0.26 | 0.02 | 0.00 | 2.2 (0.8,5.8) |
| LTC 2 | 0.01 | 0.26 | 0.63 | 0.11 | 0.00 | 1.8 (0.7,4.3) |
| LTC 3+ | 0.00 | 0.00 | 0.02 | 0.97 | 0.01 | 32.5 (25.7,41.1) |

| **Moderately obese - Ever** | Next State | | | | | Mean sojourn time in years (95% CI) |
| --- | --- | --- | --- | --- | --- | --- |
| Current state | LTC 0 | LTC 1 | LTC 2 | LTC 3+ | Death |  |
| LTC 0 | 0.85 | 0.13 | 0.02 | 0.00 | 0.00 | 5.8 (5.7,6.0) |
| LTC 1 | 0.04 | 0.74 | 0.20 | 0.02 | 0.00 | 3.0 (2.9,3.1) |
| LTC 2 | 0.00 | 0.14 | 0.70 | 0.15 | 0.00 | 2.6 (2.5,2.6) |
| LTC 3+ | 0.00 | 0.00 | 0.02 | 0.97 | 0.01 | 28.9 (27.8,30.1) |

| **Moderately obese - Resolved** | Next State | | | | | Mean sojourn time in years (95% CI) |
| --- | --- | --- | --- | --- | --- | --- |
| Current state | LTC 0 | LTC 1 | LTC 2 | LTC 3+ | Death |  |
| LTC 0 | 0.16 | 0.70 | 0.12 | 0.01 | 0.00 | 0.0 (0.0,0.0) |
| LTC 1 | 0.16 | 0.70 | 0.13 | 0.01 | 0.00 | 0.0 (0.0,0.0) |
| LTC 2 | 0.02 | 0.07 | 0.75 | 0.17 | 0.00 | 3.3 (3.2,3.5) |
| LTC 3+ | 0.00 | 0.00 | 0.02 | 0.97 | 0.01 | 32.2 (30.0,34.4) |

| **Smoking - Ever** | Next State | | | | | Mean sojourn time in years (95% CI) |
| --- | --- | --- | --- | --- | --- | --- |
| Current state | LTC 0 | LTC 1 | LTC 2 | LTC 3+ | Death |  |
| LTC 0 | 0.93 | 0.07 | 0.01 | 0.00 | 0.00 | 12.9 (12.6,13.1) |
| LTC 1 | 0.02 | 0.77 | 0.18 | 0.02 | 0.01 | 3.6 (3.4,3.7) |
| LTC 2 | 0.00 | 0.14 | 0.7 | 0.15 | 0.01 | 2.6 (2.5,2.7) |
| LTC 3+ | 0.00 | 0.00 | 0.02 | 0.96 | 0.02 | 21.8 (21.0,22.6) |

| **Smoking - Resolved** | Next State | | | | | Mean sojourn time in years (95% CI) |
| --- | --- | --- | --- | --- | --- | --- |
| Current state | LTC 0 | LTC 1 | LTC 2 | LTC 3+ | Death |  |
| LTC 0 | 0.88 | 0.10 | 0.01 | 0.00 | 0.00 | 7.9 (7.8,8.1) |
| LTC 1 | 0.04 | 0.79 | 0.16 | 0.02 | 0.00 | 3.9 (3.7,4.0) |
| LTC 2 | 0.00 | 0.12 | 0.73 | 0.15 | 0.00 | 3.0 (3.0,3.1) |
| LTC 3+ | 0.00 | 0.00 | 0.02 | 0.97 | 0.01 | 27.0 (25.9,28.1) |

| **High cholesterol ever** | Next State | | | | | Mean sojourn time in years (95% CI) |
| --- | --- | --- | --- | --- | --- | --- |
| Current state | LTC 0 | LTC 1 | LTC 2 | LTC 3+ | Death |  |
| LTC 0 | 0.32 | 0.61 | 0.07 | 0.01 | 0.00 | 0.0 (0.0,0.0) |
| LTC 1 | 0.32 | 0.61 | 0.07 | 0.01 | 0.00 | 0.0 (0.0,0.0) |
| LTC 2 | 0.03 | 0.05 | 0.77 | 0.15 | 0.00 | 3.7 (3.6,3.7) |
| LTC 3+ | 0.00 | 0.00 | 0.02 | 0.97 | 0.01 | 27.2 (26.3,28.1) |

| **Substance use*** | Next State | | | | | Mean sojourn time in years (95% CI) |
| --- | --- | --- | --- | --- | --- | --- |
| Current state | LTC 0 | LTC 1 | LTC 2 | LTC 3+ | Death |  |
| LTC 0 | 0.15 | 0.39 | 0.40 | 0.07 | 0.00 | 0.0 (0.0,0.0) |
| LTC 1 | 0.15 | 0.39 | 0.40 | 0.07 | 0.00 | 0.0 (0.0,0.0) |
| LTC 2 | 0.15 | 0.39 | 0.40 | 0.07 | 0.00 | 0.00 (0.0,0.0) |
| LTC 3+ | 0.00 | 0.01 | 0.01 | 0.97 | 0.02 | 28.6 (27.3,30.0) |

*substance use is unable to be age adjusted

Supplementary Table 4a-4j The most important antecedent and consequent associations for each LTC, with accompanying probabilities in brackets.

| **Male (n=396431)** | | | | | |
| --- | --- | --- | --- | --- | --- |
| LTC Cluster^a^ | Antecedents | LTC^b^ | Consequents | n (%) Period Prevalence of LTC | n (%) resolved |
| A | dep (0.19),ibd (0.19),ast (0.26) | anx | dep (0.51),hyp (0.05),cp (0.18) | 43285 (10.9) |  |
| C | lupus (0.39),osteoarth (0.29),scd (0.35) | cp | dep (0.15),osteoarth (0.11),anx (0.11) | 39614 (10.0) | 3992 (1.0) |
| F | ibd (0.11),ld (0.06),scd (0.08) | ast | dep (0.12),anx (0.26),cp (0.14) | 38876 (9.8) | 31317 (7.9) |
| C | dm (0.30),morbidobese (0.17),chd (0.19) | hyp | dm (0.14),ckd (0.07),cp (0.16) | 35534 (9.0) | 181 (<0.1) |
| A | ms (0.16),anx (0.51),cp (0.15) | dep | hyp (0.08),anx (0.19),cp (0.23) | 35313 (8.9) | 10219 (2.6) |
| C | hyp (0.14),morbidobese (0.16),chd (0.09) | dm | hyp (0.30),ckd (0.05),cp (0.16) | 18909 (4.8) | 88 (<0.1) |
| F | dem (0.37),osteo (0.26),can (0.31) | death |  | 14379 (3.6) |  |
| E | dep (0.07),mh (0.06),subdep (0.12) | ad | dep (0.13),anx (0.14),cp (0.14) | 13328 (3.4) | 2935 (0.7) |
| C | lupus (0.07),ra (0.08),cp (0.11) | osteoarth | dm (0.07),hyp (0.13),cp (0.29) | 12010 (3.0) |  |
| C | osteo (0.08),ckd (0.07),copd (0.07) | can | hyp (0.09),d (0.31),cp (0.15) | 11077 (2.8) | 1146 (0.3) |
| B | hf (0.06),af (0.07),pad (0.06) | chd | hf (0.09),hyp (0.19),cp (0.11) | 8115 (2.0) |  |
| E | ad (0.10),mh (0.10),vh (0.09) | subdep | dep (0.13),anx (0.15),cp (0.17) | 7884 (2.0) | 11 (<0.1) |
| B | dem (0.07),af (0.08),strktia (0.08) | strktia | hyp (0.13),d (0.13),cp (0.13) | 6981 (1.8) |  |
| B | hf (0.10),hyp (0.07),pad (0.08) | ckd | hyp (0.10),d (0.19),cp (0.14) | 6913 (1.7) | 1123 (0.3) |
| F | dep (0.07),ld (0.10),subdep (0.05) | mh | dep (0.12),anx (0.11),cp (0.14) | 6713 (1.7) |  |
| F | dm (0.04),hyp (0.03),ld (0.09) | morbidobese | dm (0.16),hyp (0.17),cp (0.16) | 6112 (1.5) | 2326 (0.6) |
| F | pad (0.05),ra (0.04),chd (0.04) | copd | hyp (0.11),d (0.12),cp (0.15) | 5594 (1.4) |  |
| E | dep (0.02),ibd (0.02),vh (0.04) | hiv | dep (0.14),anx (0.18),vh (0.13) | 5119 (1.3) |  |
| B | hf (0.09),osteo (0.06),strktia (0.05) | af | hf (0.14),d (0.13),cp (0.10) | 4692 (1.2) | 186 (<0.1) |
| F | ld (0.09),ms (0.04),strktia (0.02) | epil | dep (0.08),anx (0.11),cp (0.25) | 4451 (1.1) | 3487 (0.9) |
| D | hiv (0.13),liverdisease (0.18),subdep (0.11) | vh | liverdisease (0.13),anx (0.11),cp (0.14) | 4387 (1.1) |  |
| B | af (0.14),chd (0.09),ckd (0.05) | hf | af (0.09),d (0.18),ckd (0.10) | 3718 (0.9) |  |
| F | ms (0.03),park (<0.01),ast (0.01) | ibd | hyp (0.12),anx (0.19),ast (0.11) | 2721 (0.7) |  |
| B | osteo (0.05),park (0.09),ckd (0.04) | dem | strktia (0.07),d (0.37),cp (0.11) | 2649 (0.7) |  |
| B | chd (0.03),ckd (0.02),copd (0.02) | pad | hyp (0.11),d (0.11),cp (0.15) | 2363 (0.6) |  |
| D | ad (0.04),hiv (0.02),vh (0.13) | liverdisease | vh (0.18),d (0.14),cp (0.13) | 2248 (0.6) |  |
| F | epil (0.04),mh (0.02),scd (0.01) | ld | mh (0.10),anx (0.14),cp (0.13) | 1458 (0.4) |  |
| F | lupus (0.01),pad (<0.01),cp (0.03) | ra | hyp (0.16),osteoarth (0.08),d (0.09) | 945 (0.2) |  |
| F | dem (0.02),osteo (<0.01),ckd (<0.01) | park | dem (0.09),d (0.22),cp (0.16) | 782 (0.2) |  |
| B | dem (<0.01),park (0.02),ra (0.01) | osteo | d (0.26),can (0.08),cp (0.20) | 568 (0.1) |  |
| F | epil (<0.01),ld (<0.01),morbidobese (<0.01) | scd | hyp (0.09),ast (0.08),cp (0.35) | 447 (0.1) |  |
| F | epil (<0.01),ibd (<0.01),ast (<0.01) | ms | dep (0.16),hyp (0.10),anx (0.10),cp (0.22) | 354 (<0.1) |  |
| F | epil (<0.01),ra (<0.01),cp (<0.01) | lupus | osteoarth (0.07),anx (0.07),cp (0.39) | 112 (<0.1) |  |

| **Female (n=430434)** | | | | | |
| --- | --- | --- | --- | --- | --- |
| LTC Cluster^a^ | Antecedents | LTC^b^ | Consequents | n (%) Period Prevalence of LTC | n (%) resolved |
| A | dep (0.24),ibd (0.24),ast (0.30) | anx | dep (0.50),hyp (0.04),cp (0.23) | 73434 (17.1) |  |
| C | lupus (0.33),osteoarth (0.32),scd (0.35) | cp | dep (0.17),osteoarth (0.14),anx (0.17) | 63983 (14.9) | 4765 (1.1) |
| A | ms (0.15),anx (0.50),cp (0.17) | dep | hyp (0.07),anx (0.24),cp (0.31) | 54589 (12.7) | 14478 (3.4) |
| F | epil (0.08),ibd (0.09),scd (0.09) | ast | dep (0.14),anx (0.30),cp (0.20) | 44226 (10.3) | 33714 (7.8) |
| C | dm (0.26),ra (0.15),chd (0.16) | hyp | dm (0.11),osteoarth (0.12),cp (0.22) | 36730 (8.5) | 174 (<0.1) |
| C | hyp (0.12),ra (0.13),cp (0.14) | osteoarth | hyp (0.13),anx (0.08),cp (0.32) | 20982 (4.9) |  |
| C | hyp (0.11),morbidobese (0.12),chd (0.07) | dm | hyp (0.26),osteoarth (0.08),cp (0.17) | 16340 (3.8) | 98 (<0.1) |
| F | dm (0.06),hyp (0.06),ld (0.10) | morbidobese | hyp (0.14),anx (0.13),cp (0.23) | 13589 (3.2) | 4502 (1.0) |
| F | dem (0.34),hf (0.23),can (0.27) | death | #N/A | 12642 (2.9) |  |
| C | liverdisease (0.04),ckd (0.04),copd (0.05) | can | hyp (0.09),d (0.27),cp (0.18) | 10741 (2.5) | 1502 (0.3) |
| B | hf (0.11),osteo (0.08),pad (0.09) | ckd | hyp (0.08),d (0.18),cp (0.14) | 8252 (1.9) | 1414 (0.3) |
| B | dem (0.06),af (0.09),strktia (0.07) | strktia | hyp (0.10),d (0.15),cp (0.14) | 6467 (1.5) |  |
| E | dep (0.04),mh (0.03),subdep (0.08) | ad | dep (0.14),anx (0.22),cp (0.18) | 5995 (1.4) | 1446 (0.3) |
| F | dep (0.05),ld (0.07),subdep (0.03) | mh | dep (0.12),anx (0.13),cp (0.20) | 5099 (1.2) |  |
| B | hf (0.06),af (0.06),pad (0.06) | chd | hf (0.08),hyp (0.16),cp (0.14) | 4865 (1.1) |  |
| F | ad (0.02),pad (0.04),chd (0.03) | copd | hyp (0.10),d (0.11),cp (0.17) | 4211 (1.0) |  |
| B | osteo (0.08),park (0.11),ckd (0.06) | dem | d (0.34),ckd (0.07),cp (0.12) | 3974 (0.9) |  |
| B | hf (0.09),strktia (0.05),ckd (0.05) | af | hf (0.13),d (0.14),cp (0.10) | 3950 (0.9) | 88 (<0.1) |
| F | ld (0.07),ms (0.02),strktia (0.02) | epil | dep (0.09),anx (0.16),cp (0.27) | 3851 (0.9) | 2978 (0.7) |
| E | ad (0.09),mh (0.03),vh (0.04) | subdep | dep (0.14),anx (0.17),cp (0.19) | 3341 (0.8) | 7 (<0.1) |
| B | af (0.13),chd (0.08),copd (0.04) | hf | d (0.23),ckd (0.11),cp (0.10) | 3203 (0.7) |  |
| B | dem (0.04),park (0.04),ckd (0.04) | osteo | osteoarth (0.09),d (0.14),cp (0.20) | 3159 (0.7) |  |
| F | hiv (<0.01),scd (<0.01),ast (<0.01) | ibd | dep (0.12),anx (0.24),cp (0.17) | 3024 (0.7) |  |
| D | hiv (0.08),liverdisease (0.17),subdep (0.08) | vh | dep (0.10),anx (0.15),cp (0.20) | 2692 (0.6) |  |
| F | lupus (0.02),pad (<0.01),cp (0.05) | ra | hyp (0.15),osteoarth (0.13),anx (0.10) | 2549 (0.6) |  |
| D | ad (0.05),hiv (0.03),vh (0.09) | liverdisease | vh (0.17),d (0.11),cp (0.14) | 1443 (0.3) |  |
| B | af (0.01),chd (0.02),copd (0.02) | pad | d (0.13),ckd (0.09),cp (0.14) | 1309 (0.3) |  |
| E | scd (<0.01),subdep (<0.01),vh (0.02) | hiv | hyp (0.13),anx (0.12),cp (0.19) | 1170 (0.3) |  |
| F | epil (0.04),mh (0.01),morbidobese (<0.01) | ld | morbidobese (0.10),anx (0.15),cp (0.21) | 956 (0.2) |  |
| F | epil (<0.01),liverdisease (<0.01),ra (0.01) | lupus | hyp (0.14),anx (0.11),cp (0.33) | 803 (0.2) |  |
| F | epil (<0.01),lupus (<0.01),mh (<0.01) | ms | dep (0.15),anx (0.19),cp (0.32) | 715 (0.2) |  |
| F | hiv (<0.01),lupus (<0.01),vh (<0.01) | scd | hyp (0.09),anx (0.16),ast (0.09),cp (0.35) | 616 (0.1) |  |
| F | dem (0.01),ld (<0.01),osteo (<0.01) | park | dem (0.11),d (0.23),cp (0.19) | 542 (0.1) |  |

| **White (n=445865)** | | | | | |
| --- | --- | --- | --- | --- | --- |
| LTC Cluster^a^ | Antecedents | LTC^b^ | Consequents | n (%) Period Prevalence of LTC | n (%) resolved |
| A | dep (0.23),ibd (0.26),ast (0.32) | anx | dep (0.53),hyp (0.04),cp (0.22) | 75161 (16.9) |  |
| A | anx (0.53),ast (0.15),cp (0.18) | dep | hyp (0.06),anx (0.23),cp (0.28) | 57598 (12.9) | 14370 (3.2) |
| C | lupus (0.30),ms (0.29),osteoarth (0.31) | cp | dep (0.18),osteoarth (0.11),anx (0.15) | 55598 (12.5) | 4083 (0.9) |
| F | epil (0.06),ibd (0.10),scd (0.07) | ast | dep (0.15),anx (0.32),cp (0.15) | 51499 (11.6) | 41074 (9.2) |
| C | dm (0.24),ra (0.14),chd (0.18) | hyp | dm (0.08),osteoarth (0.09),cp (0.17) | 31846 (7.1) | 175 (<0.1) |
| C | hyp (0.09),ra (0.11),cp (0.11) | osteoarth | hyp (0.13),anx (0.07),cp (0.31) | 16802 (3.8) |  |
| F | dem (0.36),hf (0.21),can (0.27) | death | #N/A | 14734 (3.3) |  |
| E | mh (0.06),subdep (0.11),vh (0.07) | ad | dep (0.14),anx (0.18),cp (0.15) | 13785 (3.1) | 3266 (0.7) |
| C | hyp (0.06),pad (0.06),ckd (0.06) | can | hyp (0.09),d (0.27),cp (0.16) | 13194 (3.0) | 1693 (0.4) |
| C | hyp (0.08),morbidobese (0.13),chd (0.07) | dm | hyp (0.24),morbidobese (0.06),anx (0.06),cp (0.15) | 12948 (2.9) | 81 (<0.1) |
| F | dm (0.06),hyp (0.04),ld (0.10) | morbidobese | dm (0.13),hyp (0.13),cp (0.20) | 9297 (2.1) | 3441 (0.8) |
| B | hf (0.11),osteo (0.07),pad (0.08) | ckd | hyp (0.07),d (0.19),cp (0.14) | 7748 (1.7) | 1394 (0.3) |
| B | hf (0.07),af (0.07),pad (0.06) | chd | hf (0.08),hyp (0.18),cp (0.12) | 7477 (1.7) |  |
| B | af (0.08),scd (0.07),strktia (0.08) | strktia | hyp (0.11),d (0.13),cp (0.12) | 7204 (1.6) |  |
| F | pad (0.06),ra (0.04),chd (0.05) | copd | hyp (0.10),d (0.11),cp (0.16) | 7083 (1.6) |  |
| E | ad (0.09),mh (0.06),vh (0.10) | subdep | dep (0.13),anx (0.16),cp (0.17) | 6969 (1.6) | 10 (<0.1) |
| B | hf (0.10),strktia (0.05),ckd (0.05) | af | hf (0.13),d (0.13),cp (0.10) | 5903 (1.3) | 183 (<0.1) |
| F | dep (0.05),ld (0.08),subdep (0.04) | mh | dep (0.12),anx (0.14),cp (0.18) | 5620 (1.3) |  |
| F | ld (0.06),ms (0.02),scd (0.07) | epil | dep (0.09),anx (0.15),cp (0.26) | 5088 (1.1) | 3991 (0.9) |
| B | osteo (0.07),park (0.10),ckd (0.06) | dem | d (0.36),ckd (0.06),cp (0.12) | 3718 (0.8) |  |
| B | af (0.13),chd (0.08),ckd (0.05) | hf | af (0.10),d (0.21),ckd (0.11) | 3694 (0.8) |  |
| E | dep (0.01),subdep (0.02),vh (0.04) | hiv | dep (0.14),anx (0.20),vh (0.14) | 3488 (0.8) |  |
| D | hiv (0.14),liverdisease (0.09),subdep (0.12) | vh | dep (0.10),anx (0.14),cp (0.16) | 3201 (0.7) |  |
| F | ms (<0.01),scd (0.03),ast (<0.01) | ibd | dep (0.11),anx (0.26),cp (0.13) | 3082 (0.7) |  |
| B | park (0.04),ra (0.04),ckd (0.04) | osteo | d (0.16),ckd (0.07),cp (0.20) | 2778 (0.6) |  |
| B | hf (0.02),chd (0.03),copd (0.02) | pad | hyp (0.12),d (0.11),cp (0.14) | 2278 (0.5) |  |
| D | ad (0.05),hiv (0.02),vh (0.09) | liverdisease | vh (0.09),d (0.13),cp (0.15) | 2103 (0.5) |  |
| F | lupus (<0.01),osteoarth (<0.01),cp (0.04) | ra | hyp (0.14),osteoarth (0.11),anx (0.09) | 1893 (0.4) |  |
| F | epil (0.03),mh (<0.01),ast (<0.01) | ld | morbidobese (0.10),anx (0.14),cp (0.18) | 1155 (0.3) |  |
| F | dem (0.01),mh (<0.01),osteo (<0.01) | park | dem (0.10),d (0.21),cp (0.17) | 770 (0.2) |  |
| F | dep (<0.01),epil (<0.01),lupus (<0.01) | ms | dep (0.15),anx (0.17),cp (0.29) | 725 (0.2) |  |
| F | liverdisease (<0.01),ms (<0.01),ra (<0.01) | lupus | hyp (0.09),anx (0.11),cp (0.30) | 358 (<0.1) |  |
| F | ad (<0.01),hyp (<0.01),ast (<0.01) | scd | dep (0.13),d (0.13),cp (0.23) | 58 (<0.1) |  |

| **Black (n=113775)** | | | | | |
| --- | --- | --- | --- | --- | --- |
| LTC Cluster^a^ | Antecedents | LTC^b^ | Consequents | n (%) Period Prevalence of LTC | n (%) resolved |
| C | lupus (0.38),osteoarth (0.30),scd (0.36) | cp | hyp (0.14),osteoarth (0.16),anx (0.14) | 24111 (21.2) | 2222 (2.0) |
| C | dm (0.31),hiv (0.16),morbidobese (0.17) | hyp | dm (0.16),osteoarth (0.10),cp (0.23) | 23056 (20.3) | 88 (<0.1) |
| A | dep (0.18),ld (0.19),ast (0.18) | anx | dep (0.38),hyp (0.09),cp (0.22) | 16052 (14.1) |  |
| A | ms (0.21),anx (0.38),subdep (0.12) | dep | hyp (0.11),anx (0.18),cp (0.27) | 12331 (10.8) | 3124 (2.7) |
| C | hyp (0.16),morbidobese (0.14),chd (0.11) | dm | hyp (0.31),osteoarth (0.07),cp (0.19) | 11975 (10.5) | 63 (<0.1) |
| F | ibd (0.09),ld (0.07),scd (0.08) | ast | hyp (0.14),anx (0.18),cp (0.23) | 11255 (9.9) | 7383 (6.5) |
| C | hyp (0.10),ra (0.14),cp (0.16) | osteoarth | dm (0.10),hyp (0.14),cp (0.30) | 8738 (7.7) |  |
| F | ibd (0.07),ld (0.10),ast (0.07) | morbidobese | dm (0.14),hyp (0.17),cp (0.22) | 6305 (5.5) | 2184 (1.9) |
| B | hf (0.12),af (0.09),pad (0.10) | ckd | hyp (0.11),d (0.12),cp (0.15) | 4316 (3.8) | 688 (0.6) |
| F | dem (0.31),park (0.26),can (0.27) | death | #N/A | 4235 (3.7) |  |
| C | osteo (0.06),ckd (0.06),copd (0.07) | can | hyp (0.11),d (0.27),cp (0.18) | 3871 (3.4) | 234 (0.2) |
| F | dep (0.07),ld (0.09),subdep (0.07) | mh | dep (0.12),hyp (0.12),cp (0.16) | 3262 (2.9) |  |
| B | dem (0.08),af (0.09),ms (0.07) | strktia | hyp (0.13),d (0.10),cp (0.16) | 3009 (2.6) |  |
| E | dep (0.04),ld (0.03),subdep (0.10) | ad | hyp (0.12),subdep (0.11),cp (0.16) | 1998 (1.8) | 525 (0.5) |
| D | hiv (0.08),liverdisease (0.33),subdep (0.04) | vh | hyp (0.15),liverdisease (0.14),cp (0.19) | 1933 (1.7) |  |
| B | hf (0.06),af (0.05),pad (0.05) | chd | hf (0.12),hyp (0.16),cp (0.13) | 1914 (1.7) |  |
| E | ad (0.11),dep (0.03),mh (0.07) | subdep | dep (0.12),anx (0.13),cp (0.20) | 1644 (1.4) | 3 (<0.1) |
| B | af (0.16),chd (0.12),copd (0.06) | hf | d (0.16),ckd (0.12),cp (0.11) | 1553 (1.4) |  |
| E | ibd (0.04),subdep (0.01),vh (0.02) | hiv | hyp (0.16),anx (0.11),cp (0.18) | 1482 (1.3) |  |
| B | osteo (0.10),pad (0.06),park (0.10) | dem | strktia (0.08),d (0.31),cp (0.11) | 1464 (1.3) |  |
| F | ld (0.09),ms (0.04),strktia (0.03) | epil | hyp (0.08),anx (0.09),cp (0.27) | 1241 (1.1) | 838 (0.7) |
| B | hf (0.08),chd (0.04),ckd (0.03) | af | hf (0.16),d (0.15),cp (0.09) | 1089 (1.0) | 45 (<0.1) |
| F | hf (0.03),subdep (0.02),ast (0.02) | copd | dm (0.08),hyp (0.11),cp (0.17) | 979 (0.9) |  |
| F | ld (<0.01),park (<0.01),ast (<0.01) | ibd | hyp (0.12),anx (0.12),cp (0.16) | 821 (0.7) |  |
| F | ld (<0.01),ra (<0.01),vh (<0.01) | scd | hyp (0.10),anx (0.12),cp (0.36) | 740 (0.7) |  |
| D | ad (0.03),hiv (0.03),vh (0.14) | liverdisease | hyp (0.08),vh (0.33),cp (0.11) | 712 (0.6) |  |
| F | lupus (0.03),osteoarth (<0.01),cp (0.03) | ra | hyp (0.16),osteoarth (0.14),anx (0.10) | 667 (0.6) |  |
| F | epil (0.05),mh (0.02),ast (<0.01) | ld | morbidobese (0.10),anx (0.19),cp (0.14) | 665 (0.6) |  |
| B | ms (0.02),park (0.02),ckd (0.02) | pad | d (0.13),ckd (0.10),cp (0.14) | 622 (0.5) |  |
| F | ra (<0.01),subdep (<0.01),ast (<0.01) | lupus | hyp (0.16),anx (0.07),cp (0.38) | 289 (0.3) |  |
| B | dem (0.02),af (0.01),park (0.01) | osteo | dem (0.10),d (0.15),cp (0.15) | 274 (0.2) |  |
| F | dem (0.02),osteo (0.01),ra (<0.01) | park | dem (0.10),d (0.26),cp (0.17) | 197 (0.2) |  |
| F | ld (<0.01),lupus (<0.01),mh (<0.01) | ms | dep (0.21),anx (0.16),cp (0.28) | 114 (0.1) |  |

| **Asian (n=49941)** | | | | | |
| --- | --- | --- | --- | --- | --- |
| LTC Cluster^a^ | Antecedents | LTC^b^ | Consequents | n (%) Period Prevalence of LTC | n (%) resolved |
| C | lupus (0.29),osteoarth (0.30),scd (0.80) | cp | dep (0.13),osteoarth (0.16),anx (0.13) | 6381 (12.8) |  |
| A | dep (0.21),ms (0.33),ast (0.23) | anx | dep (0.47),hyp (0.06),cp (0.22) | 5606 (11.2) | 24 (<0.1) |
| C | dm (0.33),ra (0.17),chd (0.18) | hyp | dm (0.21),osteoarth (0.09),cp (0.20) | 5292 (10.6) | 3191 (6.4) |
| F | ibd (0.11),ld (0.07),ra (0.06) | ast | hyp (0.12),anx (0.23),cp (0.21) | 4576 (9.2) | 13 (<0.1) |
| C | hyp (0.21),morbidobese (0.18),chd (0.16) | dm | hyp (0.33),osteoarth (0.06),cp (0.18) | 4460 (8.9) | 818 (1.6) |
| A | hiv (0.13),scd (0.20),anx (0.47) | dep | hyp (0.08),anx (0.21),cp (0.26) | 3970 (7.9) |  |
| C | osteo (0.12),ra (0.10),cp (0.16) | osteoarth | dm (0.11),hyp (0.14),cp (0.30) | 2291 (4.6) |  |
| B | hyp (0.07),af (0.08),lupus (0.07) | chd | dm (0.16),hyp (0.18),cp (0.14) | 1318 (2.6) |  |
| F | dem (0.32),hf (0.21),can (0.22) | death | #N/A | 1220 (2.4) | 192 (0.4) |
| B | hf (0.17),af (0.09),pad (0.10) | ckd | hyp (0.10),d (0.15),cp (0.13) | 1091 (2.2) | 67 (0.1) |
| C | liverdisease (0.08),ckd (0.04),copd (0.07) | can | hyp (0.13),d (0.22),cp (0.17) | 936 (1.9) |  |
| B | dem (0.07),af (0.11),pad (0.08) | strktia | hyp (0.12),d (0.12),cp (0.17) | 803 (1.6) | 325 (0.7) |
| F | hyp (0.02),ibd (0.05),lupus (0.02) | morbidobese | dm (0.18),hyp (0.12),cp (0.20) | 748 (1.5) |  |
| F | hiv (0.08),ld (0.08),subdep (0.09) | mh | dep (0.13),dm (0.14),cp (0.17) | 645 (1.3) |  |
| D | hiv (0.15),liverdisease (0.39),subdep (0.03) | vh | liverdisease (0.22),anx (0.13),cp (0.17) | 572 (1.1) |  |
| B | af (0.15),osteo (0.06),chd (0.09) | hf | d (0.21),ckd (0.17),cp (0.11) | 477 (1.0) | 115 (0.2) |
| E | hiv (0.04),mh (0.03),subdep (0.09) | ad | dep (0.12),anx (0.14),cp (0.16) | 455 (0.9) |  |
| F | ld (0.03),pad (0.05),ast (0.04) | copd | dm (0.10),hyp (0.16),cp (0.17) | 398 (0.8) |  |
| F | epil (0.01),hiv (0.02),ld (0.01) | ibd | hyp (0.16),anx (0.19),cp (0.13) | 373 (0.7) |  |
| B | lupus (0.07),osteo (0.09),park (0.11) | dem | strktia (0.07),d (0.32),cp (0.10) | 349 (0.7) | 14 (<0.1) |
| B | hf (0.06),osteo (0.03),strktia (0.04) | af | hf (0.15),strktia (0.11),d (0.13) | 347 (0.7) | 220 (0.4) |
| F | ld (0.16),lupus (0.02),ms (0.11) | epil | dm (0.10),anx (0.12),cp (0.26) | 315 (0.6) |  |
| F | lupus (0.02),osteo (<0.01),cp (0.05) | ra | hyp (0.17),osteoarth (0.10),anx (0.13) | 278 (0.6) | 1 (<0.1) |
| E | ad (0.09),hiv (0.08),mh (0.05) | subdep | ad (0.09),mh (0.09),anx (0.13),cp (0.18) | 264 (0.5) |  |
| D | ad (0.06),hiv (0.04),vh (0.22) | liverdisease | vh (0.39),can (0.08),cp (0.13) | 251 (0.5) |  |
| B | dem (0.03),ra (0.03),ckd (0.02) | osteo | osteoarth (0.12),d (0.11),cp (0.16) | 221 (0.4) |  |
| B | chd (0.02),ckd (0.02),copd (0.02) | pad | strktia (0.08),d (0.08),ckd (0.10),cp (0.21) | 212 (0.4) |  |
| E | ad (<0.01),dem (<0.01),vh (0.02) | hiv | dep (0.13),anx (0.19),vh (0.15) | 123 (0.2) |  |
| F | epil (0.04),mh (0.01),vh (0.01) | ld | dm (0.11),epil (0.16),cp (0.18) | 122 (0.2) |  |
| F | dem (0.01),hf (0.01),ckd (0.01) | park | dm (0.13),d (0.16),cp (0.17) | 94 (0.2) |  |
| F | epil (0.01),ra (<0.01),ckd (<0.01) | lupus | dem (0.07),hyp (0.07),osteoarth (0.07),anx (0.10),chd (0.07),cp (0.29) | 75 (0.2) |  |
| F | ast (<0.01),chd (<0.01),cp (<0.01) | ms | dep (0.11),dm (0.11),epil (0.11),anx (0.33),d (0.11),cp (0.22) | 25 (<0.1) |  |
| F | dm (<0.01),lupus (0.02),anx (<0.01) | scd | dep (0.20),cp (0.80) | 11 (<0.1) |  |

| **IMD 1 – most deprived (n=144107)** | | | | | |
| --- | --- | --- | --- | --- | --- |
| LTC Cluster^a^ | Antecedents | LTC^b^ | Consequents | n (%) Period Prevalence of LTC | n (%) resolved |
| C | lupus (0.36),osteoarth (0.30),scd (0.36) | cp | dep (0.15),osteoarth (0.13),anx (0.14) | 23303 (16.2) | 2032 (1.4) |
| A | dep (0.19),ibd (0.16),ast (0.24) | anx | dep (0.46),hyp (0.05),cp (0.22) | 20860 (14.5) |  |
| A | ms (0.20),anx (0.46),cp (0.15) | dep | hyp (0.08),anx (0.19),cp (0.28) | 16670 (11.6) | 4367 (3.0) |
| C | dm (0.28),ra (0.15),chd (0.18) | hyp | dm (0.13),osteoarth (0.09),cp (0.20) | 16314 (11.3) | 60 (<0.1) |
| F | ibd (0.09),ld (0.07),scd (0.07) | ast | dep (0.12),anx (0.24),cp (0.21) | 14106 (9.8) | 10572 (7.3) |
| C | hyp (0.13),morbidobese (0.12),chd (0.09) | dm | hyp (0.28),osteoarth (0.06),cp (0.17) | 8355 (5.8) | 46 (<0.1) |
| C | hyp (0.09),ra (0.11),cp (0.13) | osteoarth | dm (0.07),hyp (0.13),cp (0.30) | 7198 (5.0) |  |
| F | dem (0.35),park (0.28),can (0.32) | death | #N/A | 6585 (4.6) |  |
| F | dm (0.05),hyp (0.05),ld (0.11) | morbidobese | dm (0.12),hyp (0.14),cp (0.22) | 4827 (3.3) | 1651 (1.1) |
| C | ms (0.06),osteo (0.06),copd (0.07) | can | hyp (0.09),d (0.32),cp (0.16) | 4345 (3.0) | 472 (0.3) |
| E | dep (0.04),mh (0.05),subdep (0.09) | ad | dep (0.12),anx (0.14),cp (0.15) | 3888 (2.7) | 862 (0.6) |
| B | hf (0.10),af (0.07),pad (0.08) | ckd | hyp (0.08),d (0.18),cp (0.14) | 3432 (2.4) | 608 (0.4) |
| B | dem (0.07),af (0.07),strktia (0.07) | strktia | hyp (0.13),d (0.15),cp (0.14) | 3113 (2.2) |  |
| B | hf (0.05),af (0.06),pad (0.08) | chd | hf (0.10),hyp (0.18),cp (0.12) | 2787 (1.9) |  |
| F | dep (0.06),ld (0.08),subdep (0.04) | mh | dep (0.10),anx (0.11),cp (0.17) | 2661 (1.8) |  |
| E | ad (0.10),mh (0.07),vh (0.07) | subdep | dep (0.13),anx (0.14),cp (0.19) | 2501 (1.7) | 5 (<0.1) |
| F | pad (0.05),park (0.04),chd (0.04) | copd | hyp (0.10),d (0.12),cp (0.15) | 2490 (1.7) |  |
| F | ad (0.03),ld (0.09),ms (0.03) | epil | dep (0.08),hyp (0.08),anx (0.13),cp (0.23) | 1769 (1.2) | 1353 (0.9) |
| B | hf (0.08),osteo (0.05),ckd (0.05) | af | hf (0.13),d (0.15),cp (0.10) | 1765 (1.2) | 47 (<0.1) |
| D | hiv (0.11),liverdisease (0.17),subdep (0.10) | vh | liverdisease (0.10),anx (0.12),cp (0.17) | 1704 (1.2) |  |
| B | af (0.13),chd (0.10),ckd (0.05) | hf | d (0.20),ckd (0.10),cp (0.09) | 1636 (1.1) |  |
| B | osteo (0.08),park (0.07),ckd (0.05) | dem | strktia (0.07),d (0.35),cp (0.09) | 1536 (1.1) |  |
| E | ibd (0.02),subdep (0.02),vh (0.03) | hiv | dep (0.11),anx (0.13),cp (0.15) | 1379 (1.0) |  |
| F | hiv (<0.01),ms (0.02),ast (<0.01) | ibd | hyp (0.11),anx (0.16),cp (0.15) | 989 (0.7) |  |
| B | chd (0.03),ckd (0.02),copd (0.02) | pad | hyp (0.09),d (0.14),cp (0.16) | 887 (0.6) |  |
| D | ad (0.04),hiv (0.02),vh (0.10) | liverdisease | vh (0.17),d (0.17),cp (0.13) | 794 (0.6) |  |
| F | lupus (0.04),park (<0.01),cp (0.04) | ra | hyp (0.15),osteoarth (0.11),anx (0.09) | 767 (0.5) |  |
| B | af (0.02),park (0.02),ra (0.03) | osteo | dem (0.08),d (0.19),cp (0.19) | 690 (0.5) |  |
| F | epil (0.03),mh (0.01),ast (<0.01) | ld | morbidobese (0.11),anx (0.14),cp (0.14) | 647 (0.4) |  |
| F | lupus (<0.01),mh (<0.01),vh (<0.01) | scd | hyp (0.08),anx (0.13),cp (0.36) | 316 (0.2) |  |
| F | dem (0.02),mh (<0.01),ckd (<0.01) | park | dem (0.07),d (0.28),cp (0.18) | 291 (0.2) |  |
| F | dep (<0.01),ld (<0.01),mh (<0.01) | ms | dep (0.20),anx (0.13),cp (0.22) | 227 (0.2) |  |
| F | epil (<0.01),ms (<0.01),ra (0.01) | lupus | hyp (0.12),anx (0.11),cp (0.36) | 198 (0.1) |  |

| **IMD 5 – least deprived (n=11390)** | | | | | |
| --- | --- | --- | --- | --- | --- |
| LTC Cluster^a^ | Antecedents | LTC^b^ | Consequents | n (%) Period Prevalence of LTC | n (%) resolved |
| A | hiv (0.33),subdep (0.29),ast (0.40) | anx | dep (0.57),hyp (0.03),ast (0.03),cp (0.24) | 1857 (16.3) |  |
| F | epil (0.09),ibd (0.13),ms (0.20) | ast | dep (0.14),anx (0.40),cp (0.14) | 1423 (12.5) | 1226 (10.8) |
| A | scd (0.50),anx (0.57),cp (0.22) | dep | hyp (0.07),anx (0.26),cp (0.35) | 1160 (10.2) | 308 (2.7) |
| C | dep (0.35),ld (0.50),ms (0.40) | cp | dep (0.22),hyp (0.10),anx (0.18) | 1046 (9.2) | 80 (0.7) |
| C | dm (0.27),park (0.22),scd (0.50) | hyp | dm (0.10),osteoarth (0.12),cp (0.14) | 634 (5.6) | 2 (<0.1) |
| C | hyp (0.12),lupus (0.33),ra (0.12) | osteoarth | hyp (0.11),ckd (0.08),cp (0.32) | 277 (2.4) |  |
| C | hyp (0.10),morbidobese (0.10),park (0.11) | dm | hyp (0.27),anx (0.09),cp (0.19) | 263 (2.3) |  |
| C | hiv (0.13),lupus (0.17),ra (0.18) | can | hyp (0.14),anx (0.14),d (0.25) | 234 (2.1) | 34 (0.3) |
| E | dep (0.05),liverdisease (0.12),subdep (0.05) | ad | dep (0.15),anx (0.22),subdep (0.15),cp (0.18) | 186 (1.6) | 52 (0.5) |
| F | ld (0.50),liverdisease (0.06),ra (0.06) | morbidobese | hyp (0.16),anx (0.22),cp (0.22) | 162 (1.4) | 70 (0.6) |
| F | hf (0.23),osteo (0.24),can (0.25) | death | #N/A | 156 (1.4) |  |
| B | af (0.11),ms (0.20),strktia (0.19) | strktia | strktia (0.19),d (0.14),cp (0.12) | 123 (1.1) |  |
| B | hf (0.13),hyp (0.06),osteoarth (0.08) | ckd | hf (0.11),hyp (0.08),osteoarth (0.08),cp (0.16) | 114 (1.0) | 18 (0.2) |
| B | pad (0.13),park (0.11),copd (0.10) | chd | hf (0.14),hyp (0.13),cp (0.12) | 110 (1.0) |  |
| F | ad (0.02),ibd (0.07),ast (0.01) | epil | dep (0.13),hyp (0.15),anx (0.13),cp (0.22) | 93 (0.8) | 79 (0.7) |
| B | pad (0.09),subdep (0.08),ckd (0.07) | af | hyp (0.15),strktia (0.11),d (0.16) | 87 (0.8) | 3 (<0.1) |
| F | anx (<0.01),ast (0.01),can (<0.01) | ibd | dep (0.13),hyp (0.13),anx (0.27),ast (0.13),cp (0.20) | 74 (0.6) |  |
| E | ad (0.15),hf (0.04),mh (0.16) | subdep | dep (0.11),anx (0.29),cp (0.13) | 70 (0.6) |  |
| F | dem (0.05),pad (0.04),chd (0.04) | copd | hyp (0.20),anx (0.14),cp (0.16) | 65 (0.6) |  |
| F | dep (0.03),park (0.11),subdep (0.03) | mh | dep (0.19),anx (0.16),subdep (0.16),cp (0.25) | 65 (0.6) |  |
| B | lupus (0.17),chd (0.14),ckd (0.11) | hf | hyp (0.09),d (0.23),ckd (0.13),cp (0.09) | 59 (0.5) |  |
| B | dem (0.16),liverdisease (0.06),ra (0.06) | osteo | dem (0.12),hyp (0.12),d (0.24),cp (0.12) | 53 (0.5) |  |
| D | hiv (0.07),liverdisease (0.12),subdep (0.08) | vh | hyp (0.12),liverdisease (0.21),cp (0.25) | 43 (0.4) |  |
| B | liverdisease (0.06),osteo (0.12),ckd (0.05) | dem | osteo (0.16),d (0.16),cp (0.21) | 41 (0.4) |  |
| D | af (0.03),subdep (0.03),vh (0.21) | liverdisease | ad (0.12),vh (0.12),can (0.12),cp (0.24) | 41 (0.4) |  |
| E | epil (0.02),mh (0.03),vh (0.04) | hiv | dep (0.13),hyp (0.13),anx (0.33),can (0.13) | 38 (0.3) |  |
| F | pad (0.04),ckd (0.01),cp (0.05) | ra | dep (0.12),hyp (0.12),osteoarth (0.12),can (0.18) | 38 (0.3) |  |
| B | hf (0.04),park (0.11),chd (0.05) | pad | hyp (0.17),af (0.09),osteoarth (0.09),anx (0.09),d (0.09),chd (0.13),cp (0.09) | 32 (0.3) |  |
| F | hyp (<0.01),ast (<0.01),cp (<0.01) | ms | hyp (0.20),strktia (0.20),ast (0.20),cp (0.40) | 18 (0.2) |  |
| F | ad (0.01),dm (<0.01),cp (<0.01) | lupus | dep (0.17),hf (0.17),osteoarth (0.33),can (0.17),cp (0.17) | 12 (0.1) |  |
| F | hf (0.02),af (0.02),strktia (0.01) | park | dm (0.11),hyp (0.22),mh (0.11),pad (0.11),anx (0.11),d (0.11),chd (0.11),cp (0.11) | 12 (0.1) |  |
| F | ast (<0.01) | ld | morbidobese (0.50),cp (0.50) | 5 (<0.1) |  |
| F | #N/A | scd | dep (0.50),hyp (0.50) | 3 (<0.1) |  |

^a^ Results from cluster analysis from Bisquera et al.^1^ A) anxiety and depression (the “mental health” cluster);

B) heart failure, atrial fibrillation, CKD, CHD, stroke/TIA, PAD, dementia, and osteoporosis (the “cardiovascular” cluster); C) osteoarthritis, cancer, chronic pain, hypertension, and diabetes (the “pain” cluster);

D) chronic liver disease and viral hepatitis (the “liver disease” cluster);

E) substance and alcohol dependency and HIV (the “dependence” cluster);

F) conditions not identified as highly correlated with any particular cluster

^b^ad= Alcohol dependency, af = Atrial fibrillation, anx=Anxiety, ast=Asthma , can=Cancer, chd=Coronary heart disease, ckd=chronic kidney disease, copd= Chronic obstructive pulmonary disease, cp=Chronic pain, , d=death, dem=Dementia, dep=Depression, dm=Type 2 diabetes, epil=Epilepsy, hf= Heart failure , hiv= human immunodeficiency virus, hyp=Hypertension,ibd= Inflammatory bowel disease, ld=Learning disability, mh=Severe mental health, morbidobese= morbid obesity, ms=Multiple sclerosis, osteo=Osteoporosis, osteoarth=Osteoarthritis, pad= Peripheral artery disease, park=Parkinsons, ra=Rheumatoid arthritis, scd=Sickle-Cell Anaemia, strktia=Stroke/TIA, subdep=Substance dependency, vh=Viral hepatitis

**Supplementary Table 5 Sensitivity analyses comparing different categorizations of the risk factor morbid obesity**

Morbid obesity (BMI ≥ 40.0 kg/m^2^) was included in the multimorbidity definition for two reasons. Firstly, the NICE Obesity guideline^2^ clearly defines morbid obesity and the medicalised approach to the management of this condition (bariatric surgery, Multi-Disciplinary Team management, medication such as Orlistat). Whereas the management of moderate obesity (BMI ≥ 30.0-39.9 kg/m^2^) is more focussed on lifestyle changes and a less biomedical model. Secondly, morbid obesity is an indicator of social disadvantage, which gives a social dimension to our definition of multimorbidity. The cut point with moderate obesity (considered a Risk Factor in this study) is inevitably going to be artificial. But to include moderate obesity as one of the included LTCs in a definition of multimorbidity would be to dilute its definition, since it is high prevalence and often gives very little functional or lifestyle impairment. It’s classification as a Risk Factor seems more justified.

However, we acknowledge that there may be bias in the use of these cut-offs particularly in presenting the differences in transition rates according to the risk factor as by definition, those who are morbidly obese will not be considered as having the moderate obesity risk factor. Therefore, we present the table below showing the hazards ratios according to the original categorization of the risk factor (a), and two more analyses showing the change in hazard ratio estimates when patients who are morbidly obese were grouped with the moderate obesity sample (b), and when the morbidly obese patients were removed from the analysis entirely (c).

|  | Progressing transitions | | | | | | | Resolving/Remitting transitions | | |
| --- | --- | --- | --- | --- | --- | --- | --- | --- | --- | --- |
|  | **0 -> 1 LTC** | **1 -> 2 LTC** | **2 -> 3+ LTC** | **0 -> Death** | **1 -> Death** | **2 -> Death** | **3+ -> Death** | **1-> 0 LTC** | **2 -> 1 LTC** | **3+ -> 2 LTC** |
| a) Ever moderate obese vs none and morbid obese (n=826870) | 2·95 (2·88, 3·03) | 1·75 (1·69, 1·81) | 1·29 (1·26, 1·32) | 0·20 (0·11, 0·37) | 0·29 (0·24, 0·36) | 0·36 (0·28, 0·45) | 0·58 (0·56, 0·61) | 1·61 (1·53, 1·69) | 1·40 (1·33, 1·48) | 0·78 (0·74, 0·83) |
| b) Ever moderate and morbid obese vs none (n=826870) | 3.01 (2.94, 3.09) | 1.72 (1.66, 1.78) | 1.32 (1.28, 1.35) | 0.19 (0.10, 0.37) | 0.29 (0.24, 0.36) | 0.37 (0.29, 0.46) | 0.59 (0.57, 0.61) | 1.62 (1.55, 1.7) | 1.41 (1.34, 1.49) | 0.79 (0.75, 0.84) |
| c) Ever moderate obese vs none (morbid obesity removed from the sample)(n=807455) | 2.56 (2.50, 2.63) | 1.56 (1.50, 1.62) | 1.15 (1.12, 1.18) | 0.21 (0.11, 0.38) | 0.42 (0.34, 0.51) | 0.25 (0.19, 0.32) | 0.62 (0.60, 0.65) | 1.35 (1.28, 1.42) | 1.23 (1.15, 1.31) | 0.64 (0.59, 0.68) |

**Supplementary Fig 1: Summary of resolve logic applied to each condition**


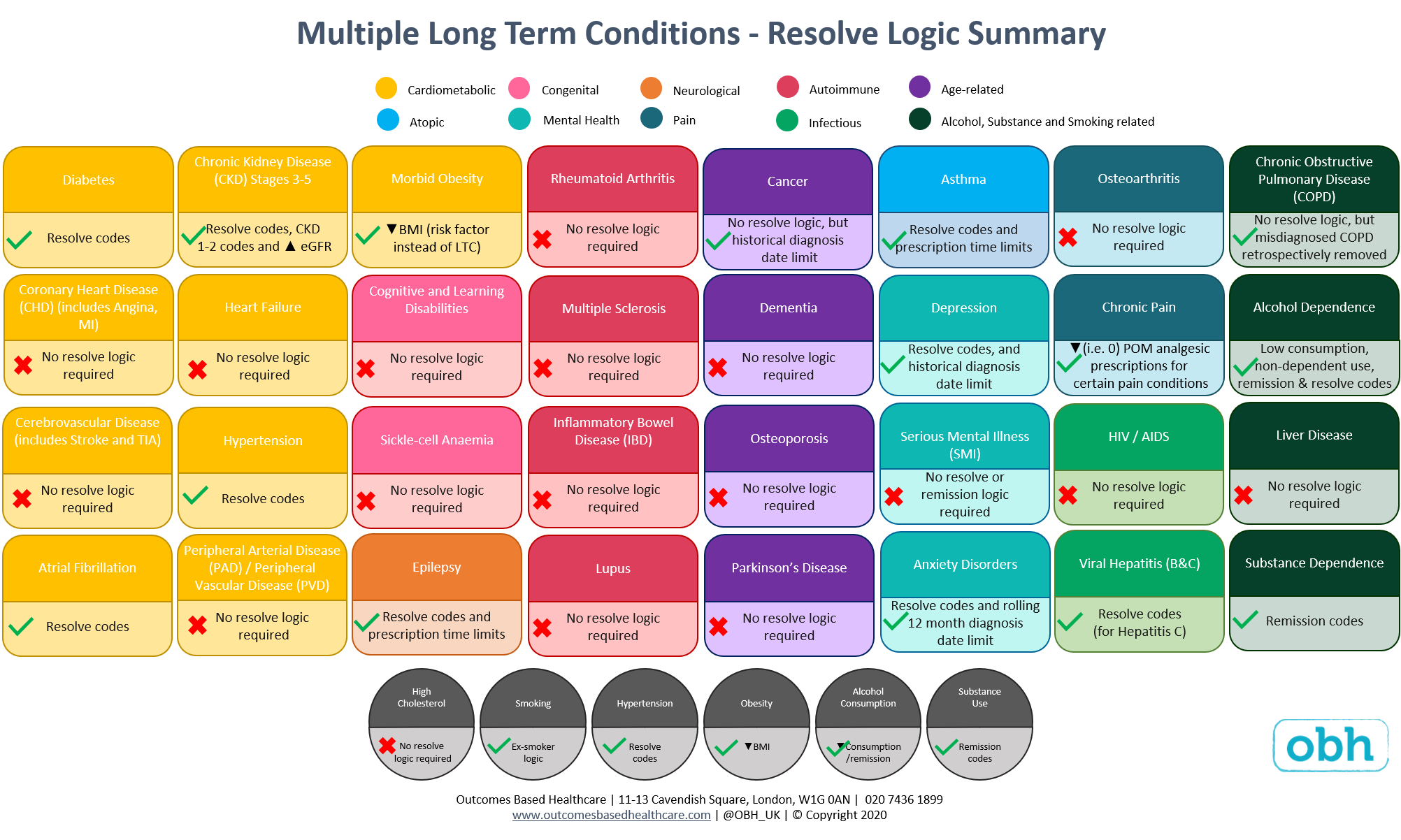


*Source: Outcomes Based Healthcare. 2020. Summary of chronic condition resolve logic, developed in collaboration with King’s College London*

Supplementary Fig 2: Representation of the first order Markov chain to model probabilities of acquiring three conditions can be inflated to model 32 conditions as per our study. Markov chains are represented by the transition matrix (right side of the figure), where each element of the matrix is the probability of moving from one condition to another from time n to time n+1. Graphs such as that of the left-hand side of the figure can enable a better understanding of this transition matrix. For example, the probability of developing condition B given the person has condition A is 0.30, while the probability of the development from condition B to A is 0.15, while the probability of remaining with condition B from one timepoint to the next is 0.65.

0.5

0.50 0.30 0.20

P = 0.15 0.65 0.20

0.15

0.30

B

0.65

0.6

0.20

0.20

0.20

0.50 0.30 0.20

P = 0.15 0.65 0.20

0.20 0.20 0.60

A

0.20

C

**Supplementary Fig 3:** **Comparison of mortality rates between the Lambeth Datanet (LDN) and deaths registered in England and Wales (E&W). Mortality rates have been age standardized to the 2013 European Standard Population, expressed per 100,000 population.**


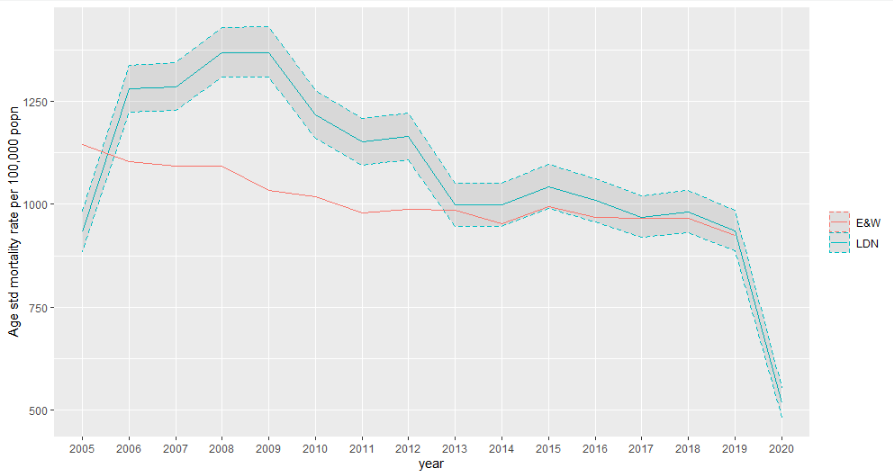


References

1. Bisquera A, Gulliford M, Dodhia H, et al. Identifying longitudinal clusters of multimorbidity in an urban setting: A population-based cross-sectional study. The Lancet Regional Health - Europe. DOI: 10.1016/j.lanepe.2021.100047.

2. National Institute for Health and Care Excellence. Obesity: identification, assessment and management (2014, accessed Aug 20, 2021).

stylefix
